# Supplementary material for: Portable molecular diagnostic platform for rapid point-of-care detection of mpox and other diseases
Source: Nat Commun. 2025 Mar 24;16:2875. doi: 10.1038/s41467-025-57647-3 (PMC11933461; doi:10.1038/s41467-025-57647-3)
Supplement: Supplementary file 1 — Suppplementary Information [file 41467_2025_57647_MOESM1_ESM.pdf]

Portable molecular diagnostic platform for rapid point-of-care detection of mpox and other diseases

Matthew L. Cavuto[1,2,&], Kenny Malpartida-Cardenas[1,2,&], Ivana Pennisi[1,2], Marcus J. Pond[3], Sohail Mirza[1,2], Nicolas Moser[2,4], Mark Comer[2], Isobel Stokes[5], Lucy Eke[5], Sian Lant[5], Katarzyna M. Szostak[2], Luca Miglietta[1], Oliver W. Stringer[1,2], Katerina-Theresa Mantikas[2,4], Rebecca P. Sumner[5], Frances Bolt[1,2], Shiranee Sriskandan[1,6], Alison Holmes[1,7,8], Pantelis Georgiou[2,4], David O. Ulaeto[9], Carlos Maluquer de Motes[5], Jesus Rodriguez-Manzano[1,2,*]

[1] Department of Infectious Disease, Faculty of Medicine, Imperial College London, London, UK
[2] ProtonDx Ltd, Translation & Innovation Hub, Imperial College London, London, UK
[3] Department of Infection and Immunity, Imperial College Healthcare NHS Trust, London, UK
[4] Department of Electrical and Electronic Engineering, Imperial College London, London, UK
[5] Department of Microbial Sciences, School of Biosciences and Medicine, University of Surrey, Guildford, UK
[6] Centre for Bacterial Resistance Biology, Imperial College London, London, UK
[7] David Price Evans Infectious Diseases & Global Health Group, University of Liverpool, Liverpool, UK
[8] The Fleming Initiative, Imperial College London and Imperial College Healthcare NHS Trust, London, UK
[9] CBR Division, Defence Science and Technology Laboratory, Salisbury, UK
[&] These authors contributed equally
[*] Corresponding author: j.rodriguez-manzano@imperial.ac.uk

Table of Contents

Supplementary Table 1. Monkeypox diagnostics landscape, as identified by FIND 2

Supplementary Figure 1. Standard curves of LAMP assays used in this study 3

Supplementary Table 2. Sequences of final LAMP assays used in this study 4

Supplementary Table 3. Summary of the performance of the LAMP assays used in this study 5

Supplementary Table 4. Synthetic DNA sequences used in this study 6

Supplementary Table 5. Input concentrations for sample-to-result analytical sensitivity performance 7

Supplementary Figure 2. Virucidal activity of eNAT® against VACV and HSV-1 8

Supplementary Figure 3. Confusion matrices illustrating clinical diagnostic performance 9

Supplementary Figure 4. Distribution of RNase P C_t values obtained from all clinical samples 10

Supplementary Figure 5. Dragonfly platform shown stored in portable backpack 11

Supplementary Table 6. PCR primer/probe sequences used in this study 12

Supplementary Methods 13

Supplementary Figure 6. Experimental design of the virucidal activity 13

Nucleic Acid Extraction Efficiency 14

Platform Robustness 15

Supplementary Table 7. Summary of preliminary robustness results 16

Android application 17

Dragonfly OPXV/MPXV Multi-patient Instructions for Use 18

Supplementary Table 1. Monkeypox diagnostics landscape, as identified by FIND.

Product-Instrument (Company)	Target	Chemistry	Time-to-result	Storage conditions	Size (mm)* Weight (kg)*	Automated workflow	Reference
Xpert® Mpox - Cepheid GeneXpert® Systems (Cepheid)	MPXV clade II and non-variola OPXV	Extracted, PCR	>36 minutes	2-28°C	161 x 305 x 297<br>25	Yes, hands-on time <1 min	https://cepheid.widen.net/s/qmwltddz29/cepheid-xpert-mpox-datasheet-us-ivd-0998-english
QIAstat-Dx Viral Vesicular Panel - QIAstat-Dx Analyzer 1.0 (QIAGEN)	MPXV Clade I and II, VZV, HSV-1, HSV-2, HHV6 and EV	Extracted, PCR	~ 70 minutes	15-25°C	234 x 326 x 517<br>21	Yes	https://www.sciencedirect.com/science/article/pii/S1386653223001488
u-card dx monkeypox virus test (Wondfo Biotech)	MPXV Clade I and II	Extracted, PCR	<40 minutes	2-30°C	315 x 245 x 355<br>11	Yes	https://en.wondfo.com/pt/index116.html
EasyNAT® Monkeypox Virus Assay - EasyNat system (Ustar)	N/A	Extracted, PCR	~ 1 hour	2-8°C	390 x 300 x 470<br>15	Yes, hand-on time <5 min	https://en.bioustar.com/product/152.html
Cue Mpox Molecular Test (Cue Health)	MPXV Clade I and II	Direct, Proprietary Isothermal NAAT	~ 25 minutes	15-30°C	74 x 74 x 37<br><1	Yes	https://cuehealth.com/products/mpox-monkeypox-molecular-test
Pluslife Monkeypox Virus Card (Pluslife)	N/A	Direct, Proprietary Isothermal NAAT	~ 35 minutes	15-30°C	101 x 91 x 65<br><1	Yes, hand-on time < 5 min	https://www.pluslife.com/companyfile/15.html
Skin Infection Viral Test Panel – Dragonfly (ProtonDx)	OPXV, MPXV clade I and II, VZV, HSV-1, and HSV-2	Extracted LAMP	<40 minutes	Room temperature<br>(Max range: -20-30°C)	160 x 110 x 130<br><1	No, hands-on time <5 min	This study

*Values provided for primary reusable equipment, for example, an accompanying automated device or in the case of the Dragonfly Platform, the isothermal heat block.

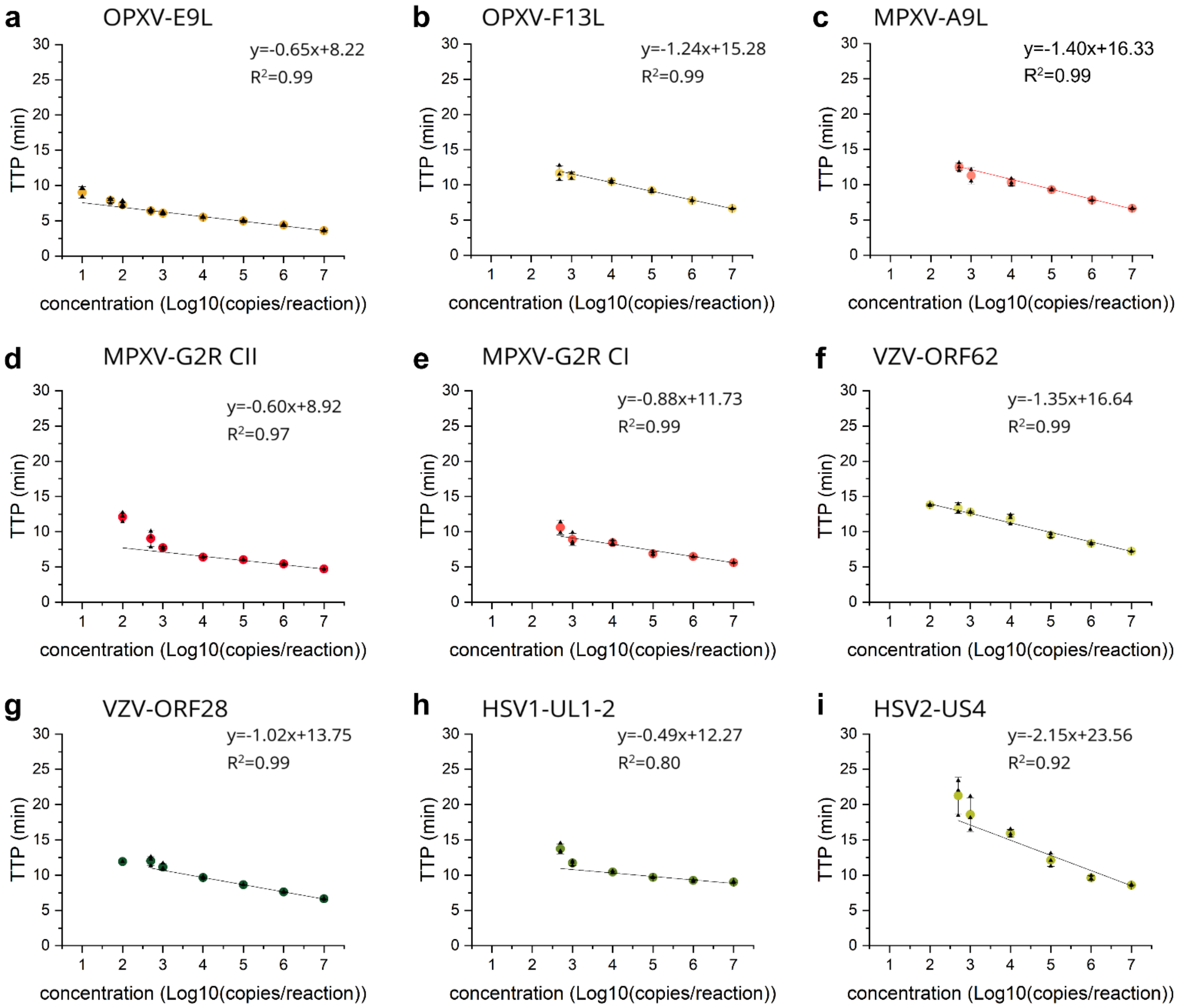

Supplementary Figure 1. Standard curves of LAMP assays used in this study. The assay LAMP-MPXV_G2R was used with synthetic DNA from MPOX Clade II (d) and Clade I (e). Replicates n=3.

Supplementary Table 2. Sequences of final LAMP assays used in this study.

Target	LAMP assay ID	Sequence (5' to 3')
OPXV	LAMP-OPV-E9L-F3	AACATTTTTGGCAGAGAGAG
	LAMP-OPV-E9L-B3	ATAGATGGCCTTTTCAGTTGAAC
	LAMP-OPV-E9L-LF	GCTAAGAGTTGCACATCCATAGG
	LAMP-OPV-E9L-LB	GATAACTCTGCTCCATTTAGTACC
	LAMP-OPV-E9L-FIP	ATCTTGTACGTATA**Y**TGCATGGAATC-AAAGATGYTAAAACAGGCTACC
	LAMP-OPV-E9L-BIP	AGTGCTCTATACTCATACGCTTCG-TCTAGATACAAKATCATTCTACGTCC
	LAMP-OPV-F13L-F3	ATTTGGCCATAGTTCCCAC
	LAMP-OPV-F13L-B3	CRTCGTCGACTATYAACAAT
	LAMP-OPV-F13L-LF	ACTCCTCTATTAATGGCTGCTT
	LAMP-OPV-F13L-LB	CCGCCAGAAGTCTAGACGC
	LAMP-OPV-F13L-FIP	CCAATTACCRACTARAAGTCTGA-ATTGGCCYGACATTTACAAC
	LAMP-OPV-F13L-BIP	AACGACGTATATTCTATGGCAAC-ACCTTCACAGATAGATCATTTTGRA
MPXV	LAMP-MPXV-G2R-F3	ACGAAAGACTGGATCACAATC
	LAMP-MPXV-G2R-B3	TCTAAAACAAAGTGTGGAATAGG
	LAMP-MPXV-G2R-LF	CACATCGTGTACTCGGAC
	LAMP-MPXV-G2R-LB	GAAGAGACGGTGTGAGAATATG
	LAMP-MPXV-G2R-FIP	GATGTGGAAATTAACCTGTATCCAGTC-GACGTTGAGATGGATTCGC
	LAMP-MPXV-G2R-BIP	GTATTGCTGGTTACGACGGG-ACGTCATCTGTTCTCCGTG
	LAMP-MPXV-G2R-BIP2	GTATTGCTGGTTACGGGTTC-ACGTCATCTGTTCTCCGTG
	LAMP-MPXV-A9L-F3	GATTGATATCGCATAGAAATAGAAAAG
	LAMP-MPXV-A9L-B3	CAGCACAAGATCRACCCC
	LAMP-MPXV-A9L-LB	CGTTTGGTAATGGCAATGTATTAAG
	LAMP-MPXV-A9L-FIP	TGTATGAACAGTACTTTGTCGATG-CTCATCATTGAAGATTACTCTGTTAC
	LAMP-MPXV-A9L-BIP	GATGAGATGTTTATATGTTGGCATAGTAG-GATGCTGCTAGACATTATGGAG
VZV	LAMP-VZV-ORF62-F3	TCAGAAGCCTCACATCCTCC
	LAMP-VZV-ORF62-B3	CGTACTGTACCCCCGAAAC
	LAMP-VZV-ORF62-LF	AGTGGAGGCGCTGCGACGGA
	LAMP-VZV-ORF62-LB	CGCAGGGCGCCAGGCCGTGG
	LAMP-VZV-ORF62-FIP	GACGGTTTGGTCCACCCAGC-TCTGGGATCTGCCGCATC
	LAMP-VZV-ORF62-BIP	GGCGCCGGGATCAAAGCTTA-GGTCGACGACCCATTGTTTC
	LAMP-VZV-ORF28-F3	CCAATACGACCACCGGATC
	LAMP-VZV-ORF28-B3	GGACTGGCTTCGTCTCG
	LAMP-VZV-ORF28-LF	CGAAATGTAGGATATAAAGG
	LAMP-VZV-ORF28-LB	CATCGGGAATAACATCCTTATATTC
	LAMP-VZV-ORF28-FIP	CTCCCACTGGTACGTCAAGTG-AGGGTCAAAAACCCTGGC
	LAMP-VZV-ORF28-BIP	CATCTCTTCCTCAACATCCCCG-TACCCGATGGGGGATACC
HSV-1	LAMP-HSV1-UL1-2-F3	CAGCCACACACCTGTGAA
	LAMP-HSV1-UL1-2-B3	TCCGTCGAGGCATCGTTAG
	LAMP-HSV1-UL1-2-LF	AAATCCTGTCGCCCTACACAGCGG
	LAMP-HSV1-UL1-2-LB	CACCCCGCGACGGGACGCCG
	LAMP-HSV1-UL1-2-FIP	CCAGACGTTCCGTTGGTAGGTC-ACTTTGACTATTCGCGCACC
	LAMP-HSV1-UL1-2-BIP	CCATCATCGCCACGTCGGAC-TCGGCGTCTGCTTTTTGTG
HSV-2	LAMP-HSV2-US4-F3	CCGTCAGCCCATCCT
	LAMP-HSV2-US4-B3	GCCCACCTCTACCCAC
	LAMP-HSV2-US4-LF	GTCTTTGGGGACGGCG
	LAMP-HSV2-US4-LB	GCGGAGACATTCGAGTACC
	LAMP-HSV2-US4-FIP	CCGCCCTGGTACGTGTA-AGTATGGAGGGTGTCGC
	LAMP-HSV2-US4-BIP	TCGTAAATGCTTCCCTGCTG-TCGCCGCCGAGTTC

Supplementary Table 3. Summary of the performance of the LAMP assays used in this study.

Assay _ID	FIP/BIP (uM per reaction)	Target	Gene	LOD (copies/reaction)	TTP (min)	Ref.
LAMP-OPV_E9L	2	OPXV	E9L	10	< 10	This study
LAMP-OPV_F13L	3	OPXV	F13L	500	< 15	This study
LAMP-MPXV_G2R	2	MPXV	G2R	100	< 15	This study
LAMP-MPXV_A9L	3	MPXV	A9L	500	< 15	This study
LAMP-VZV_ORF62	4	VZV	ORF62	100	< 15	Okamoto et al. 2004
LAMP-VZV_ORF28	2	VZV	ORF28	100	< 15	This study
LAMP-HSV1_UL1	2	HSV1	UL1-2	500	< 15	Kaneko et al. 2005
LAMP-HSV2_US4	3	HSV2	US4	500	<25	This study

Supplementary Table 4. Synthetic DNA sequences used in this study. The name indicates the target and the gene (in the case of MPXV_G2R, also the clade). This was used to investigate assay specificity of both clades, as shown in Supplementary Fig. 1.

Name	Sequence ID	Sequence
gblock_MPXV_G2R_cI	NC_003310	ATAAAACCGTATTATACTCGTATATATTGTTTCTCTCATGTATAATAATAAACGGAAGAGATTTAGCACCACATGCACCATCCAAT<br>GGAAAATGTAAAGACAACGAATACAGAAGCCGTAATCTATGTTGTCTATCGTGTCCTCCGGGAACTTACGCTTCCAGATTATGTG<br>ATAGCAAGACTAATACACAATGTACGCCGTGTGGTTCGGATACCTTTACATCTCACAATAATCATTTACAGGCTTGTCTAAGTTGT<br>AACGGAAGATGTGATAGTAATCAGGTAGAGACGCGATCGTGTAACACGACTCACAATAGTTACCCAAATTTTTTCAAAGGATCA<br>TCAGGGTGTAGAACATGTATTTCTAAAACAAAGTGTGGAATAGGATACGGAGTATCCGGATACACGTCTACCGGAGACGTCATC<br>TGTTCTCCGTGTGGTCCCGGAACATATTCTCACACCGTCTCTTCCACAGATAAATGCGAACCCGTAACCAGCAATACATTTAACTA<br>TATCGATGTGGAAATTAACCTGTATCCAGTCAACGACACATCGTGTACTCGGACGACCACTACCGGTCTCAGCGAATCCATCTCA<br>ACGTCGGAACTAACTATTACCATGAATCATAAAGATTGTGATCCCGTCTTTCGTGCAGAATACTTCTCTGTCCTTAATAATGTACG<br>AGATTAAATGTAACAACAAAGATTCAAACGAAAACAAAGAATGATACTATCATCTATATAGTAATACCAATACTCAA
gblock_MPXV_G2R_cII	NC_063383	ATTTTTTCCGTATTATACTCGTATATATTGTTTCTCTCATGTATAATAATAAACGGAAGAGATATAGCACCACATGCACCATCCAAT<br>GGAAAGTGTAAAGACAACGAATACAGAAGCCGTAATCTATGTTGTCTATCGTGTCCTCCGGGAACTTACGCTTCCAGATTATGTG<br>ATAGCAAGACTAATACACAATGTACACCGTGTGGTTCGGATACCTTTACATCTCACAATAATCATTTACAGGCTTGTCTAAGTTGT<br>AACGGAAGATGTGATAGTAATCAGGTAGAGACGCGATCGTGTAACACGACTCACAATAGTTACCCAAATTTTTTCAAAGGAGCA<br>TCAGGGTGTAGAACATGTATTTCTAAAACAAAGTGTGGAATAGGATACGGAGTATCCGGATACACGTCTACCGGAGACGTCATC<br>TGTTCTCCGTGTGGTCCCGGAACATATTCTCACACCGTCTCTTCCACAGATAAATGCGAACCCGTCGTAACCAGCAATACATTTAA<br>CTATATCGATGTGGAAATTAACCTGTATCCAGTCAACGACACATCGTGTACTCGGACGACCACTACCGGTCTCAGCGAATCCATC<br>TCAACGTCGGAACTAACTATTACCATGAATCATAAAGATTGTGATCCAGTCTTTCGTGCAGAATACTTCTCTGTCCTTAATAATTTT<br>CGAGATTAAATGTAACAACAAAGATGTTAACGAAAACAAAGAATGATACTATATACTATATAGTAATACCAATACT
gblock_MPXV_A9_MPXV	NC_063383	AAAAATTGTTAACGGTGTATTAAGCAGACAAGATTTTGATAATCTTATAGGTGTTAGACAATATATAACAGCACAAGATCAACCC<br>CGCTTTGACATCACTTATAACATCGCAGATGCTGCTAGACATTATGGAGTTAATCTTAATACATTGCCATTACCAAACGTCGATCT<br>CACTACTATGCCAACATATAAACATCTCATCATGTATGAACAGTACTTTGTCGATGATTATGATAGAGTACCAATTTATTACAATG<br>GTAACAGAGTAATCTTCAATGATGAGATTATAAACTTTACTATTTCTATGCGATATCAATCTCTTATTCCTAGACTGGTAGATTTCT<br>TTCCAGATATACCAGTAAACAATAACATCGTACTTCATACTCGCGATCCTCAA
gblock_OPV_F13L	NC_006998	AACACCTATTGGGATATTCTAGAGATCTAGATACCGATGTAGTTATTGATAAACTCAAGTCGGCTAAGACTAGTATAGATATTGA<br>ACATTTGGCCATAGTTCCCACTACACGTGTCGACGGTAATAGCTACTATTGGCCCGACATTTACAACTCCATTATAGAAGCAGCC<br>ATTAATAGAGGAGTTAAGATCAGACTTCTAGTTGGTAATTGGGATAAGAACGACGTATATTCTATGGCAACCGCCAGAAGTCTA<br>GACGCGTTGTGTGTTCAAAATGATCTATCTGTGAAGGTTTTCACTATTCAGAATAATACAAAATTGTTGATAGTCGACGACGAAT<br>ATGTTCATATCACTTCGGCAAATTTCGACGGAACCCATTACCAAAATCACGGATTCGTCAGTTTTAATAGTATAGATAAACAGCTT<br>GTAAGCG
gblock_OPV_E9L	NC_006998.1	GGAACCATTCCTAGACTATTAAGAACATTTTTGGCAGAGAGAGCCAGATATAAAAAGATGTTAAAACAGGCTACCAGTTCAACT<br>GAAAAGGCCATCTATGATTCCATGCAATATACGTACAAGATAGTAGCCAACTCAGTATATGGTCTGATGGGATTTAGAAATAGT<br>GCTCTATACTCATACGCTTCGGCTAAGAGTTGCACATCCATAGGACGTAGAATGATCTTGTATCTAGAATCGGTACTAAATGGAG<br>CAGAGTTATCTAACGGTATGTTACGGTTTGCCAATCCATTAAGTAATCCATTTTATATGGACGATAGAGATATTAATCCGATTGTG<br>AAAACATCGTTGCCTATAGATTACAGATTTCGTTTTCGTAGCGTACTAGTGAAACTAGAAGAGATGTTTCCAAGTTTCATAAGAA<br>TATGATTAAGACATACAAGACCAGACTGTCTGAGATGTTGTCTGAAGGACGGATGAATTCTAATCAGGTATGTATAGATATTCTC<br>CGTTCTTTAGAAACAGATTTACGATCCGAATTTGATAGTAGATCGTCTCCTCTAGAATTATTTATGTTGAGTCGAATGCATCACTC<br>AAATTATAAATCCGCAGATAACCCTAATATGTATTTGGTTACTGAATATAATAAAAATAATCCAGAAACTATAGAACTTGGAGAA<br>CGATATTATTTTGCATATATTTGTCCGGCTAATGTACCATGGACCAAAAAACTTGTAAATATTAAAACATATGAAACAATTATCGA<br>TAGAAGTTTTAAACTCGGCAGTGATCAAAGAATATTTTACGAAGTTTACTTTAAACGATTGACGTCCGAAATAGTCAATC
gBlock_VZV_ORF62	NC_001348	GTCCCCCTCGGGATGGACTCCATGACGGTCCCGGATCTGTCGCGAGGGTGCTCTCGAGGGGGCCGTTGATGTCCTCTCCGGGCA<br>ACGGATCGTAGATGATCAGAAGCCTCACATCCTCCGGGTCTGGGATCTGCCGCATCCAGGCGCACCTCCGTCGCAGCGCCTCCA<br>CTCCGCTGGGTGGACCAAACCGTCGGTCTCCTCCGCCCGGACGCCGAGCGGCGATTTCCGCCAAGGCGCCGGGATCAAAGCTTA<br>GCGCAGGGCGCCAGGCCGTGGGAAACAATGGGTCGTCGACCAGACGGGCGATGGTTTCGGGGGTACAGTACGCCTTGCGAGC<br>CTGGTCCGACGGGACCGGGGTATGCAGGGCCCCCCGGGGAATACGCCGAAATCCCCCGTTTGGGGCCGGTCCGTCAAGTGGCA<br>TCGTTATTACGGCGGGG
gBlock_VZV_ORF28	NC_001348	GCGCTCTAATAGCCTTGCGCATAGCCAACCAGTCTTTTAAAAGAACACCCAGCAGACTTTCTCGAACGTTAGAGCGCACAAAAAA<br>AAGACGTTTTCCTCCAACTGTAAAGGTGGCATAATCGGATGGATTCAAACGTTTAACCGTCTCAAAATTTAACGTTAGCGTGGTA<br>AAACATAAGTTATGGGCCTGAATTATACTTGGATATAAACTTGCAAAATCCAATACGACCACCGGATCGATATAAAATCCCGTAT<br>CAGGGTCAAAAACCCTGGCTCCTTTATATCCTACATTTCGCCCACTTGACGTACCAGTGGGAGAAACGCTCTCGTCTTCATCCATC<br>TCTTCCTCAACATCCCCGACATCGGGAATAACATCCTTATATTCAAAAGTAGCTGGGTATCCCCCATCGGGTAAAATAAATCCTCG<br>AGACGAAGCCAGTCCTAATAAACAGGTGTAAATCCTAACCTGCTGTCCGTCGTAAATAGCCTTGGTTAAAGTAATTCTAGCTAGC<br>CTTGCAACCGCGGATAACTCAAGGTGTGGTAAATATTTAAAAAACAGTTTCCCCACAAGAGCCGAGTCTTGTATACAATATTCAC<br>CAATAATTCCTCGTGTATTCGGTCCACTAGCGTAATATCCCGGAATGTCTTTGTAGGGCAAATCTCTCTTGGACTCATTTAGAGCT<br>TCACGTGCAACCGAATCTAATTTATAACTCGAGAGTTTTAATTTTTCAGTTGCAATTGCATACATATCCAGAGATATGAGACCGTT<br>GATCTTTACCTTGCTTCGTCGCTGAAATCCGGATTTGCCAACATCCCATATCTTAAACAGACCCCCACGGTTTATACTGCCATAAC<br>CATCAAGCTTGAGACTGTATATAGAATTAAGTTTCTC
gblock_HSV1_UL1-2	OP297860	GAAACAGAAACGCGCTTGGCCCTTTATAAAGAGATACGCCAGGCGCTGGACAGTCGCAAGCAGGCCGCCAGCCACACACCTGT<br>GAAGGCTGGGTGTGTGAACTTTGACTATTCGCGCACCCGCCGCTGTGTAGGGCGACAGGATTTGGGACCTACCAACGGAACGT<br>CTGGACGGACCCCGGTTCTGCCGCCGGACGATGAAGCGGGCCTGCAGCCGAAGCCCCTCACCACGCCGCCGCCCATCATCGCCA<br>CGTCGGACCCCACCCCGCGACGGGACGCCGCCACAAAAAGCAGACGCCGACGACCCCACTCCCGGCGCCTCTAACGATGCCTC<br>GACGGAAACCCGTCCGGGTTCGGGGGGCGAAC
gblock_HSV2_US4	NC_001798	CTGGTTTTGCTGGCGCCGCCGGTACGCGGATTTGGCGCACCCAACGCAACGTATGCGGCCCGTGTGACGTACTACCGGCTCACC<br>CGCGCCTGCCGTCAGCCCATCCTCCTTCGGCAGTATGGAGGGTGTCGCGGCGGCGAGCCGCCGTCCCCAAAGACGTGCGGGTC<br>GTACACGTACACGTACCAGGGCGGCGGGCCTCCGACCCGGTACGCTCTCGTAAATGCTTCCCTGCTGGTGCCGATCTGGGACCG<br>CGCCGCGGAGACATTCGAGTACCAGATCGAACTCGGCGGCGAGCTGCACGTGGGTCTGTTGTGGGTAGAGGTGGGCGGGGA

Supplementary Table 5. Input concentrations of viral particles into Dragonfly for sample-to-result analytical sensitivity performance.

MPXV Viral particles			HSV1/VZV Viral particles			HSV2 Viral particles		
Tube ID	Copies per mL	Copies per reaction	Tube ID	copies per mL	copies per reaction	Tube ID	Copies per mL	Copies per reaction
Tube 1	5.00E+04	2.00E+03	Tube 1	2.00E+04	8.00E+02	Tube 1	2.87E+04	1.15E+03
Tube 2	1.00E+04	4.00E+02	Tube 2	7.00E+03	2.80E+02	Tube 2	1.00E+04	4.01E+02
Tube 3	7.00E+03	2.80E+02	Tube 3	2.50E+03	1.00E+02	Tube 3	3.58E+03	1.43E+02
Tube 4	2.50E+03	1.00E+02	Tube 4	1.25E+03	5.00E+01	Tube 4	1.79E+03	7.17E+01
Tube 5	1.25E+03	5.00E+01	Tube 5	7.50E+02	3.00E+01	Tube 5	1.08E+03	4.30E+01
Tube 6	7.50E+02	3.00E+01						

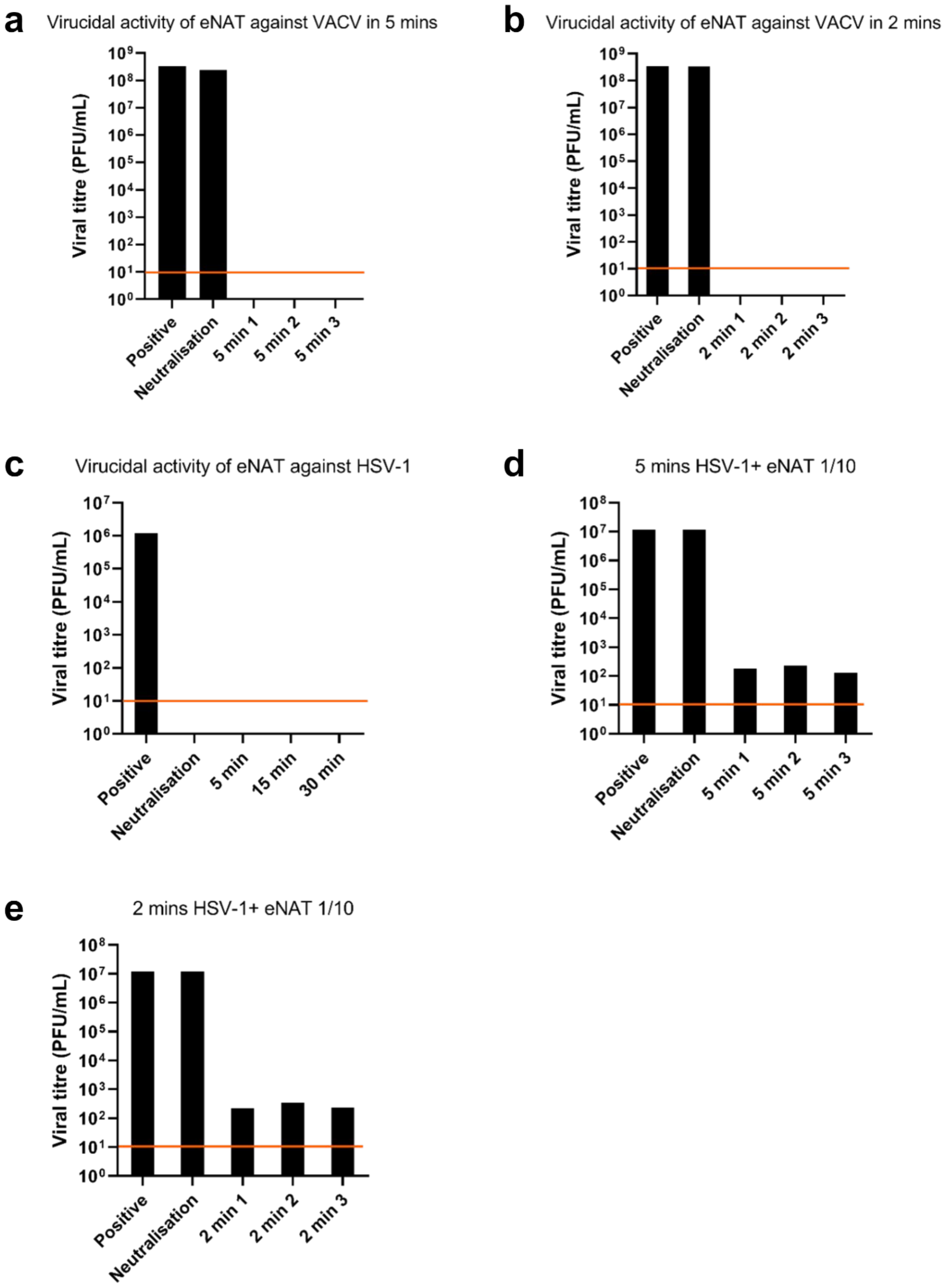

Supplementary Figure 2. Virucidal activity of eNAT® against VACV and HSV-1. Replicates n=3.

a

OPXV		PCR POS	PCR NEG
DragonFly	POS	49	0
DragonFly	NEG	2	113
	TOTAL	51	113
	SEN(%)	96.1(CI: 86.5-99.5)	
	SPE(%)	100 (CI: 96.8-100.0)	

b

MPXV		PCR POS	PCR NEG
DragonFly	POS	48	0
DragonFly	NEG	3	113
	TOTAL	51	113
	SEN(%)	94.1(CI: 83.8-98.8)	
	SPE(%)	100 (CI: 96.8-100.0)	

c

VZV		PCR POS	PCR NEG
DragonFly	POS	9	10
DragonFly	NEG	1	144
	TOTAL	10	154
	SEN(%)	90.0 (CI: 55.5-99.8)	
	SPE(%)	93.5 (CI: 88.4-96.8)	

d

HSV-1		PCR POS	PCR NEG
DragonFly	POS	18	7
DragonFly	NEG	2	137
	TOTAL	20	144
	SEN(%)	90.0 (CI: 68.3-98.8)	
	SPE(%)	95.1(CI: 93.5-99.3)	

e

HSV-2		PCR POS	PCR NEG
DragonFly	POS	7	4
DragonFly	NEG	3	150
	TOTAL	10	154
	SEN(%)	70.0 (CI: 34.8-93.3)	
	SPE(%)	97.4 (CI: 93.5-99.3)	

Supplementary Figure 3. Confusion matrices illustrating clinical diagnostic performance.

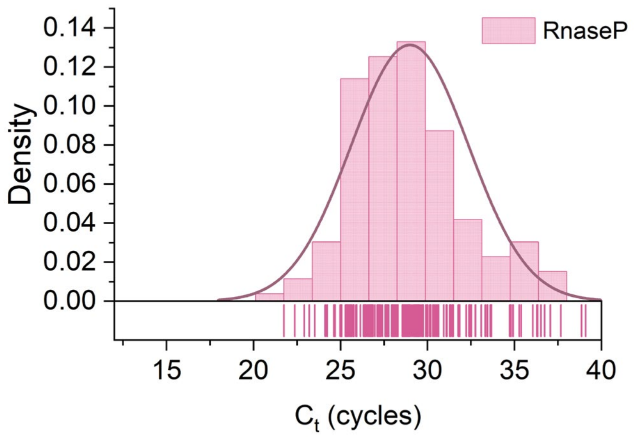

Supplementary Figure 4. Distribution of C_t values obtained from all clinical samples, using a TaqMan assay specific for RNaseP.

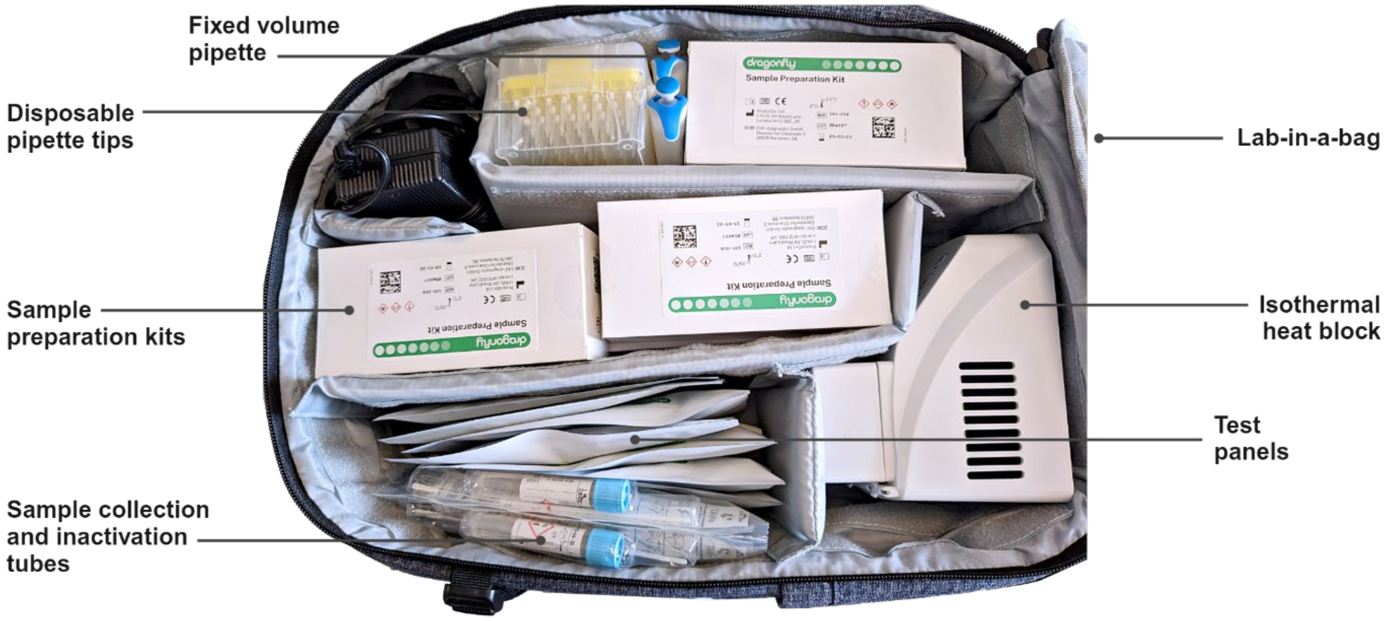

Supplementary Figure 5. Lab-in-a-bag. The entire Dragonfly platform stored in an easily portable backpack (29 x 16 x 43 cm), ready for remote deployment. Created in BioRender. Cavuto, M. (2025) https://BioRender.com/h56q657

Supplementary Table 6. PCR primer/probe sequences used in this study.

PCR assay ID	Sequence (5′ to 3′)	Reference
HSV1-F	CGGCCGTGTGACACTATCG	Weidmann et al., 2003
HSV1-R	CTCGTAAAATGGCCCCTCC	
HSV1-P	FAM-CCATACCGACCACACCGACGAACC-TAMRA	
HSV2-F	CGCTCTCGTAAATGCTTCCCT	
HSV2-R	TCTACCCACAACAGACCCACG	
HSV2-P	FAM-CGCGGAGACATTCGAGTACCAGATCG-TAMRA	
VZV-F	CGGCATGGCCCGTCTAT	
VZV-R	TCGCGTGCTGCGGC	
VZV-P	FAM-ATTCAGCAATGGAAACACACGACGCC-TAMRA	
MPXV-WA-F	CACACCGTCTCTTCCACAGA	Li et al., 2010
MPXV-WA-R	GATACAGGTTAATTTCCACATCG	
MPXV-WA-P	FAM-AACCCGTCGTAACCAGCAATACATTT-BHQ1	
OPXV-E9L-F	TCAACTGAAAAGGCCATCTATGA	Li et al., 2006
OPXV-E9L-R	GAGTATAGAGCACTATTTCTAAATCCC	
OPXV-E9L-P	Cy5-CCATGCAATATACGTACAAGATAGTAGCCAAC-BHQ2	

SUPPLEMENTARY METHODS

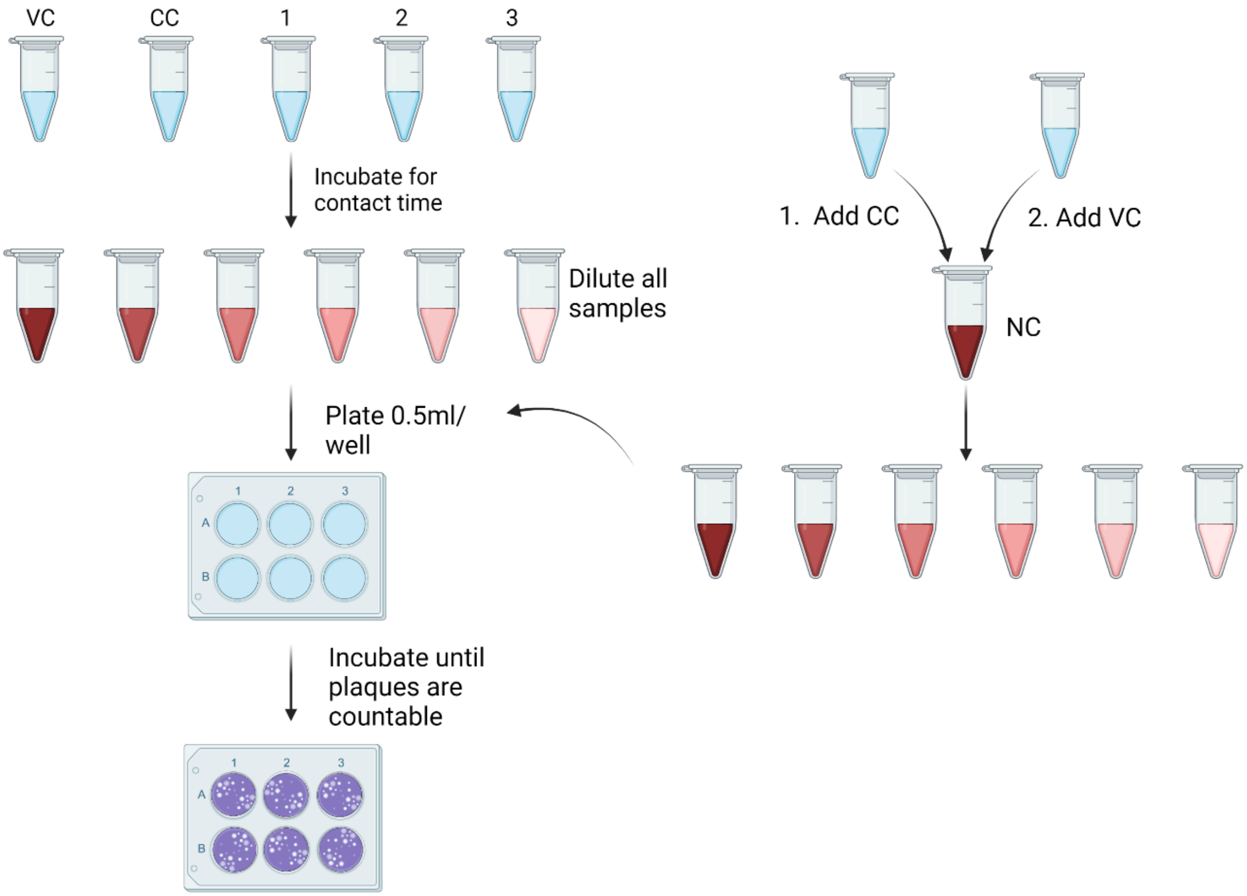

Supplementary Figure 6. Experimental design of the virucidal activity of eNAT against VACV and HSV-1. Created in BioRender. Stokes, I. (2025) https://BioRender.com/h55u092

Nucleic Acid Extraction Efficiency

To evaluate the nucleic acid extraction efficiency of the SmartLid protocol for MPXV and ensure the repeatability of the procedure, a total of 12 eNAT samples were spiked with a known concentration of MPXV viral particles (Vircell, MBTC032-R), at a final concentration of 100,000 copies per millilitre. The estimated total recovery was 37.5% (SD = 7.7%). All samples were extracted following the SmartLid protocol described in the Workflow section of the main manuscript. Overall recovery efficiency percentage was estimated by testing the eluted nucleic acids by dPCR, each eluted samples was tested in triplicate. A total of 2 µl of GoTaq Probe qPCR Mastermix (Promega Corporation, USA), 0.4 µl of 20X GE Sample Loading Reagent (Fluidigm PN 85000746), 1.76 µl of PCR grade water, 0.4 µl of 10X PCR primer mixture containing the pan MPXV qPCR primer set described in **Supplementary Table 6** (4 µM of forward primer, 4 µM of reverse primer and 2 µM of hydrolysis probe), and 1 µl of sample eluate, to bring the final volume to 4 µl. PCR cycling condition consisted of a hot start step for 2 m s at 95° C, followed by 45 cycles at 95°C for 15 s and 60° C for 30 s. We used the Integrated Fluidic Circuit controller MX (IFC) to prime and load the qdPCR 37K chips and the Fluidigm Biomark HD system to perform the thermal cycling and imaging, in accordance with manufacturer's instructions. Each digital chip contains 48 inlets, where each inlet is connected to a microfluidic panel consisting of 770 partitions or wells: each of 0.85 nL volume.

Platform Robustness

As introduced in the main manuscript, the Dragonfly platform has been preliminarily evaluated for robustness under a variety of suboptimal operating conditions. For each condition tested, eNAT® medium was spiked with a 3×LOD concentration of inactivated SARS-CoV-2 viral particles, with a "pass" requiring 3/3 positive replicates. First it was deemed important to assess the stability of nucleic acids in the elution after extraction. Accordingly, experiments were run at three different temperatures (4°C, 20°C, and 30°C) and three different time points (3, 10, and 30 minutes) per temperature. The 4°C elution tubes were stored in the 4°C cold room in our laboratories. The 20°C elution tubes were stored at room temperature. Finally, the 30°C elution tubes were stored in a GS Biotech 170L incubator at 30°C. All temperature and time points were 100% successful, enabling the claim of nucleic acid stability in elution for at least 30 minutes at 30°C. Note, while further time-points and temperatures could have been tested, it was deemed not desirable to allow users to wait longer than this from a risk and workflow perspective.

Next, due to the possibility that a user may open the foil Test Panel packaging early, exposing the strips to the outside environment, the following test was carried out to determine the maximum length of time after which the test panel could become unusable, due to likely lyophilized reagent moisture absorption and degradation. Time points of 10, 30, and 60 minutes were evaluated, after which the Respiratory Test Panels were resuspended with extracted 3×LOD samples, 30 minutes was determined to be the recommended maximum period of time after opening a test panel before it is resuspended. Similarly, it was important to anticipate a user opening the individual flip-caps of the test panel tubes prematurely, perhaps while extracting the sample, for example. Accordingly, the lyophilized reagents were exposed to air for varying time points (2, 5, and 15 minutes) at room temperature (20°C) with all tested replicates being successful. Accordingly, while longer time points could have been evaluated, it was validated that it was acceptable to leave the tube strip open, with contents still lyophilized, for at least 15 minutes. The same time points were evaluated a second time, however this time with the test panel reagents rehydrated with elution. As expected, due to the lack of long-term room temperature stability of the LAMP reagents in liquid form, results of this experiment demonstrated a maximum recommended waiting period after test panel rehydration of up to 5 minutes. Fortunately, the included Dragonfly Heat Block is able to heat up from room-temperature to the set temperature of 63.5°C in less than that period of time, covering for the scenario that a user forgets to turn on the Heat Block prior to running a test.

Next, it was critical to evaluate the performance of Dragonfly at varying operating temperatures. To remain consistent with the recommendations ascertained from the previously described experiments, each operating temperature tested (4, 20, and 30°C) was evaluated while following the maximum waiting times for each tested "worst-case-scenario" above (i.e. waiting 15 minutes after removing test panels from their packaging, opening their tubes, waiting another 15 minutes, rehydrating the reagents, and finally waiting 5 minutes before placing the rehydrated test panels in the heat block.) Results showed a viable operating temperature range for Dragonfly of 4-30°C, as. Note, operating temperatures were achieved through the same means as the first presented stability experiment, with all components first equilibrating to that temperature, before running the entire experiment in that environment from sample-to-result.

While the Dragonfly Heat Block was developed to be firmware locked at the ideal incubation temperature of 63.5°C, a ±1°C range was also evaluated, in order to account for slight variations in the heat block calibration, or fluctuations throughout heating. All tested replicates were successful. Given that ±1°C exceeds the manufacturers stated temperature accuracy of ±0.3°C, this ensured us that heat block calibration and temperature accuracy should not affect Dragonfly test performance.

One common metric for qualitative diagnostics tests (including antigen, antibody, and molecular) is end-point stability, meaning the length of time that the result is still clearly and accurately readable for, after the test is finished. For example, lateral flow strip based diagnostic tests have notoriously short end-point stability, as capillary and evaporative effects tend to smear and blur the result indication lines. In contrast, after evaluating n=15 Dragonfly Respiratory Test Panel results for all targets for six days (stored at room temperature, or roughly 20°C), all colorimetric results were still clear and easy to read.

Finally environmental nucleic acid contamination, which can result in false-positive test results, is a common issue that faces molecular diagnostic tests due to their high sensitivity. While it is possible for this contamination to come from entirely external sources, it is also possible for a running or completed test to contaminate future results. For example, in the context of the Dragonfly system, if one of the flip-cap lids on the test panel were to open in the middle of incubation, amplicons could be released into the air that could cause false-positives in ongoing adjacent and future tests. Therefore, it is important to evaluate the likelihood of this occurring. One way to do this is by utilizing a series of alternating "high-positive" and negative samples, run in quick succession, and ensuring that all results are either true-positives or true-negatives. Accordingly, a total of 10 tests were performed on eNAT® samples, half of which were spiked with 10,000 copies/mL of SARS-CoV-2 viral particles, and the other half

of which were negative. All negative samples tested were correctly identified, demonstrating a low risk of cross-contamination when performing a Dragonfly test, even when processing positive and negative samples in close succession and physical proximity.

Supplementary Table 7. Summary of preliminary robustness results.

Parameter	Result
Stability of elution	4-30°C for ≤ 30 minutes
Stability of closed test panel	≤ 30 minutes
Stability of open test panel	≤ 15 minutes
Stability of rehydrated test panel	≤ 5 minutes
Operating temperature	4-30°C
Incubation temperature	63.5°C ±1°C
End-point stability of colourimetric result	≤ 6 days
Cross-contamination	Zero demonstrated cross-contamination when alternating high-positive and negative samples (n=10)

Further work is planned to expand upon the tested ranges and tailor the examined conditions to the Skin Infection Viral Test Panel. For example, a wider operating temperature range should be tested in order to ensure applicability to the POC in warmer climates, and interfering substances likely to be found in the relevant sample type (i.e. skin lesion swabs) should be considered.

Android application

The Dragonfly application (illustrated in Fig. 3 and Fig. 5 of the main manuscript) is a tool to support the use of the Dragonfly Kit for molecular detection of a panel of infectious diseases. The Application provides step-by-step instructions to guide the user through the workflow of the Dragonfly Kit, prompts the user with test results and synchronizes the results to a cloud server.

The backend or the cloud software has been designed as an API layered system which is behind a Virtual Private cloud. The cloud software is served via the NGINX web server. The Authentication system has been provided by Auth0 and the backend syncs with the Auth0 service for handling users. The VPC is accessed by Route 53 Domain service from AWS.

The Mobile Application is broken into two parts for achieving consistency in UI across platforms with respect to future scalability: Android and React JS layers. The Android Layer handles all device level functionality and OS level consistencies. All the business logic resides in the Android layer as well. Android runs a native server which loads the React application. This layer also handles the authentication via Auth0 and runs services for synchronization of test data. The React JS application handles all the UI components. Since the UI has been thought of being consistent throughout platforms, this strategy helps in avoiding duplicity and maintaining consistency of the codebase. All the user-facing functionality shall be implemented in the React UI layer and the application will be customized to work in different UI sizes (predominantly the Tablet). React shall be using Redux states to handle state machines in the UI. All the data communication will also be synchronized with the Android layer for consistency.

The Dashboard is an SPA built on React JS. The authentication/authorization process is again linked to the Auth0 service which handles user authentication. The Dashboard renders customized UI based on the User access levels defined previously in User Roles and Responsibilities.

The Dragonfly Android application source code is available at https://github.com/nmoserpdx/dragonfly-skin-infection .

Additionally, we have designed a Progressive Web App (PWA) for a smartphone (illustrated in Fig. 5). The app aims to enhance productivity, time management and daily routines, offering partially customizable timers on the same screen and compatibility across different devices. The PWA source code is available at https://github.com/bahp/pwa-timerhub .

1. Introduction

Dragonfly comprises two unique technologies that enable high quality molecular diagnostics at the point-of-care: **SmartLid Sample Preparation** and **Colourimetric LAMP Detection**.

SmartLid is a novel method for nucleic acid extraction, centring around a proprietary magnetic lid to transfer DNA and RNA through three simple steps: Lysis, Wash, and Elution. The procedure is based on magnetic separation and utilizes the fastest collecting superparamagnetic beads on the market. See SmartLid in action below:

1. Magnetic Beads capture nucleic acids

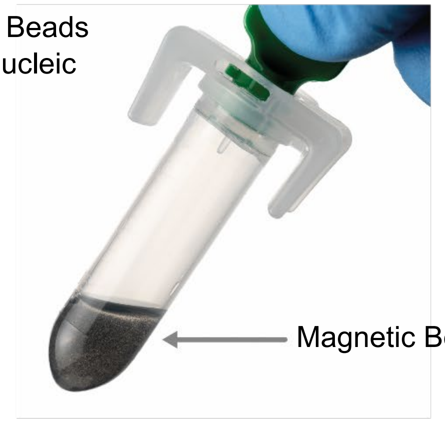

2. Inverting the tube with the Magnet inserted collects the Magnetic Beads onto SmartLid

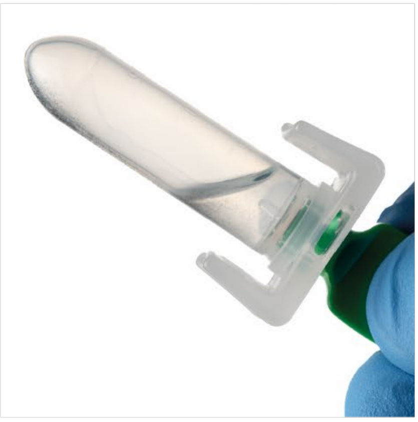

3. Within seconds, the liquid is clear and collection is complete

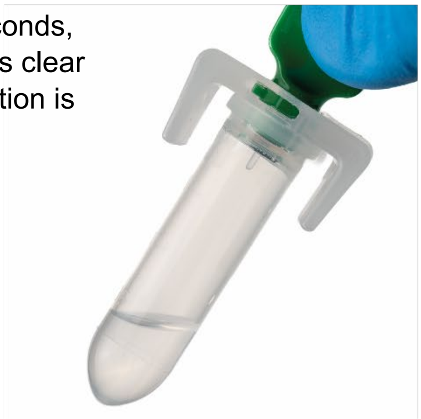

4. Magnetic Beads are safely attached to SmartLid and ready for transfer into the next tube

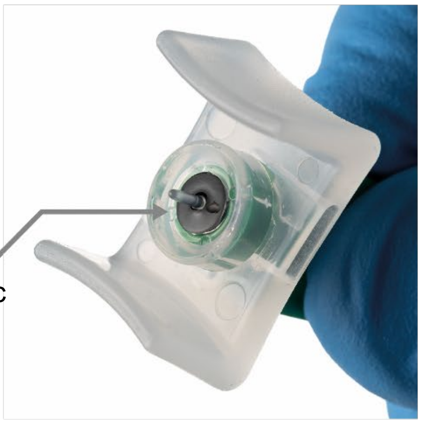

Next, Dragonfly's detection technology combines lyophilised LAMP reagents with easy-to-read colourimetric indicators, enabling room temperature storage and a virtually equipment free molecular workflow. Simply add the extracted sample, incubate, and visually read the result. Reagents start out pink when rehydrated, and turn yellow to indicate a positive result:

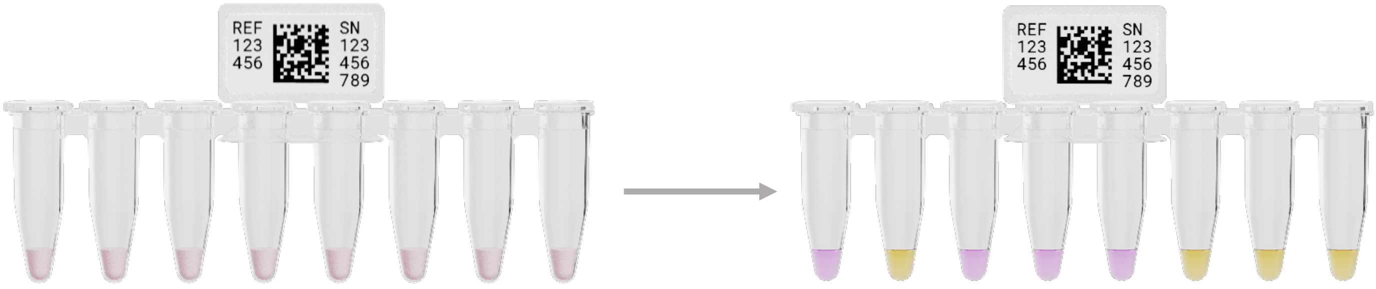

2. Safety Information

Caution	All chemicals and biological material should be considered potentially hazardous.Specimens are potentially infectious and should be treated accordingly.
	eNAT™ collection tube and tube A contain Guanidine-thiocyanate. Please ensure tubes are sealed prior to disposal, as when combined with bleach, Guanidine-thiocyanate can react to produce a highly toxic gas.
	Tube B contains ethanol which is highly flammable and can cause skin irritations.
	ProtonDx Heat block contains a hot surface, prolonged contact may cause burns.
	When working with this kit use appropriate PPE.
	After use, components should be disposed of using appropriate routes, in compliance with local regulations.
	Aerosol-barrier pipette tips are recommended for pipetting the sample elution (tube C). The pipette tips must be discarded between test kits.

Warning 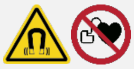	Magnetic fields can be harmful to pacemaker wearers.

Important	Ensure all Dragonfly test panel lyophilised assays are near the bottom of the Test Panel reaction tubes prior to rehydration to avoid cross contamination events and ensure effective incubation heating.
	Ensure all reaction tubes are not damaged or cracked prior to use.
	Reaction tubes should be kept closed at all times following reconstitution and discarded without opening following use, according to local health and safety guidelines. To avoid any contamination with the amplified product, never open a Dragonfly Test Panel tube during or after amplification.

3. Storage Information

All components of the Dragonfly OPXV/ MPXV Starter Kit should be stored dry at room temperature (15–25°C). If any Sample Preparation Kit components show signs of leakage, or Test Panels show signs of moisture ingress, dispose of appropriately and contact customer support.

4. Included Materials

Refer to the kit part number below for the specific configuration and contents supplied.

Kit	100367	Dragonfly OPXV / MPXV Starter kit	
**Qty**	**PN**	**Item**	**Description**
3	100065	Sample Preparation Kit bulk pack 40	Consumable
4	100067	OPXV / MPXV Test Panel bulk pack 15	Consumable
4	100088	50 x eNat Collection Swab	Consumable
2	100084	Pipette tip boxes (96 tips each)	Consumable
2	100004	Reusable Pipette	Reusable
2	100003	Heat Block	Reusable
1	100218	Vortex Mixer	Reusable
2	100365	Dual Tube Holder	Reusable

Kit	100349	Sample Preparation Kit (detailed contents)
**Qty**	**Item**	**Description**
2	400 µL disposable pipettes	Used to transfer exact volume from Sample tube into Tube A.
1	Tube A Lysis	Contains Lysis/binding buffer (≤ 61% isopropanol and ≤ 25.6% guanidinium thiocyanate) and magnetic beads.
1	Tube B Wash	Contains Wash buffer (≤ 80% ethanol).
1	Tube C Elution	Contains Elution buffer.
1	SmartLid	Includes green magnetic key and transparent base.
1	Sample Preparation tray	Workstation for holding materials during test preparation process.
1	Absorbent pad	For transport only.

Kit	100346	OPXV / MPXV Test Panel (Detailed contents)
**Qty**	**Item**	**Description**
1	Test panel	8-tube strip containing lyophilised reagents and attached identification tag.
1	Result card	Used to capture the result with the companion application, or tointerpret manually.
2	Desiccant	For transport only.

5. Protocol for the Detection of OPXV and MPXV with the Dragonfly Platform – Visual Guide

Training video:

Important Before Starting

1. Always wear PPE, such as a lab coat and gloves and change as required according to local guidelines.

2. Prior to performing any biological procedure, ensure the working environment is clean using a decontamination spray according to local guidelines.

3. Ensure the Heat Block is turned on and reads 63.5 C.

   Note: The switch on the back must be switched to the “on” position, **AND** the power button on the front of the Heat Block must be pressed for it to begin heating up.

Workstation Setup

While you are welcome to tailor your workstation to suit your preferred workflow, we recommend setting up the Dragonfly platform as shown below, with either one or two Heat Blocks:

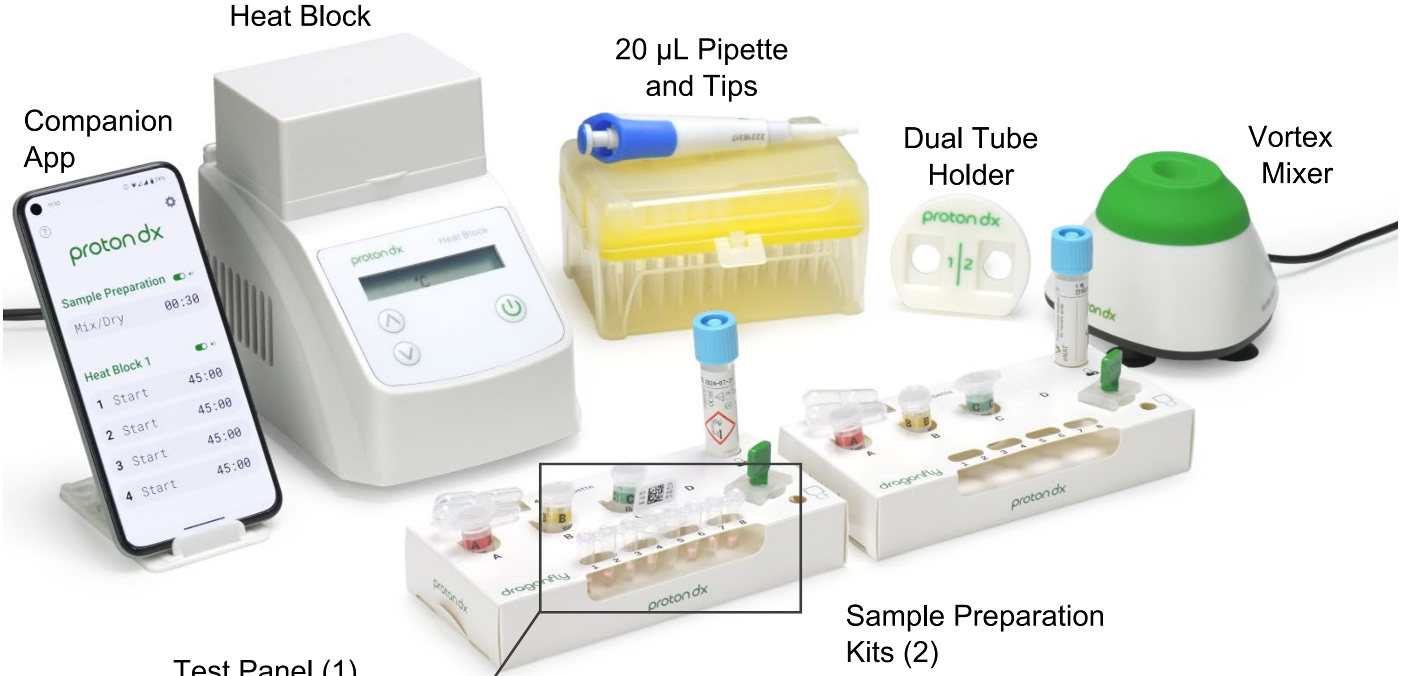

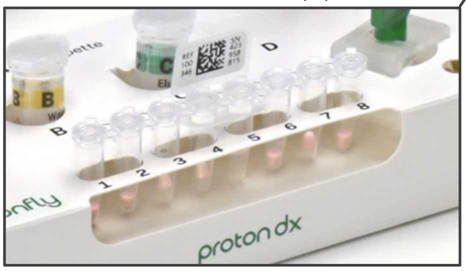

As shown, for every two Sample Preparation Kits, there is a single corresponding Test Panel. This is because every Dragonfly OPXV / MPXV Test Panel can simultaneously test two extracted samples.

We recommend starting with the Test Panel in the leftmost Sample Preparation Kit to avoid confusion when loading the panel. (See “Detection: Test Panel Loading”)

Companion Application

Access the app here:

At multiple points throughout the Dragonfly process, timing of steps is required. The Dragonfly Companion App assists with this by providing visual countdown timers with audible alarms and interactive features to limit user errors. The app runs in any web browser, and can be run on any phone, tablet, or computer, across both Android, Apple, and PC devices. See below for a summary of key features:

Note: The timers will still run while the app is in the background or if the device falls asleep. <u>However, the app must remain in the foreground, with the device awake, for the audible alarms to sound.</u>

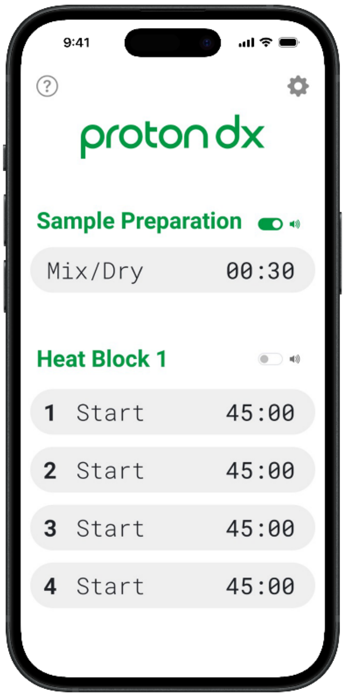

There are two timer sections, one for Sample Preparation, and another for Heat Block incubation.

Audible alarms can be toggled on and off for each timer section.

Scrolling down will reveal a second Heat Block section for processing more samples at a time.

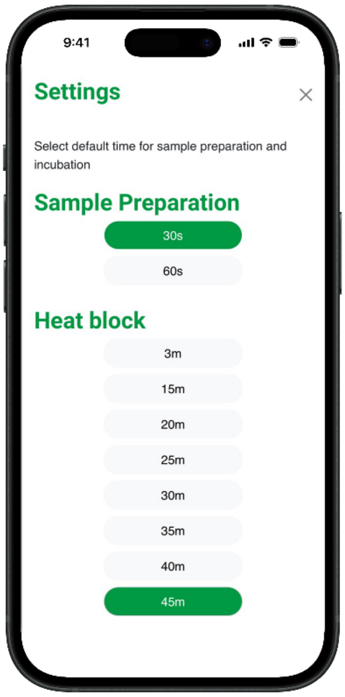

In the settings menu, select the appropriate timers for both Sample Preparation and Heat Block incubation.

For this version of Dragonfly, select **30s** and **45m** as shown.

Note: you cannot change these settings if a timer is currently active.

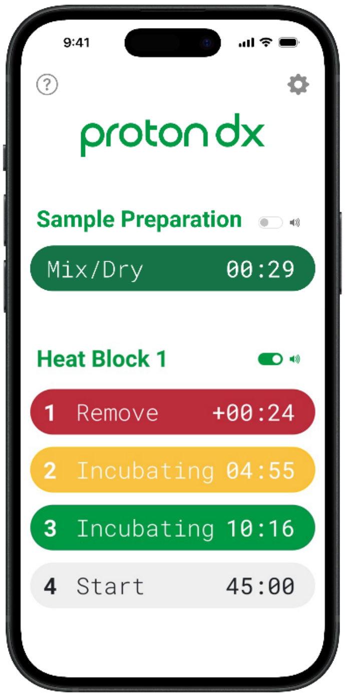

The colour of each Heat Block timer will turn yellow when 5m remain, and red when the incubation is complete.

An alarm will then sound, and the time will start counting up.

Important: Remove the test panel before the timer reaches +05:00.

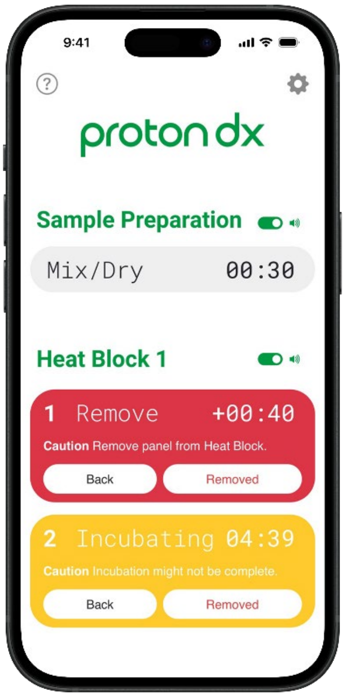

Tapping on an active Heat Block timer will display a different message depending on the time remaining.

To reset a timer, press “Removed” after removing the respective Test Panel.

A final confirmation will be required before the timer fully resets.

Processing Two Samples at a Time

This version of Dragonfly was designed to simultaneously extract and test two samples at a time. To assist with the multiple Magnetic Bead mixing and collection steps throughout the DNA Extraction process, a Dual Tube Holder is included with this kit. Its operation is described below:

Vortexing Two Samples:

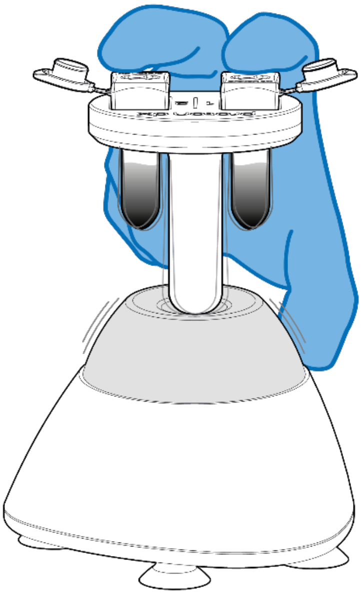

As shown, it is important that all mixing steps are conducted with the green magnet **removed** from both SmartLids.

To prevent the tubes from coming loose or falling out during mixing, we recommend covering the top of each SmartLid as demonstrated. Pressing down will then activate the Vortex Mixer.

Finally, make note of which side of the Holder is used for each sample. The top is labelled “1” and “2” to help avoid accidentally mixing them up.

Note: In the following sections and diagrams, this symbol (right) will be displayed for steps where use of the Dual Tube Holder is required if processing two samples at a time.

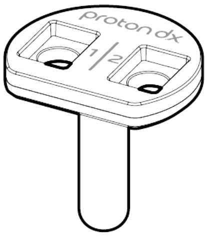

Magnetic Bead Collection for Two Samples:

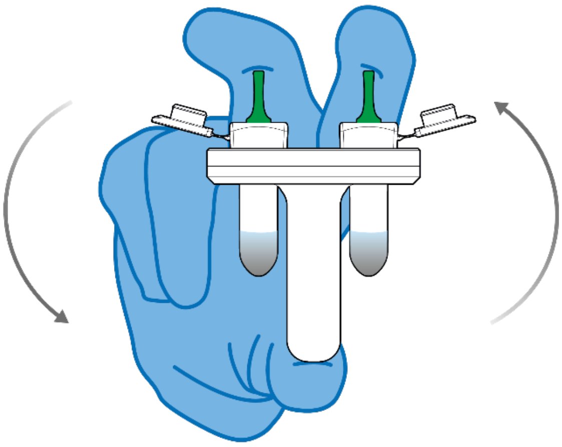

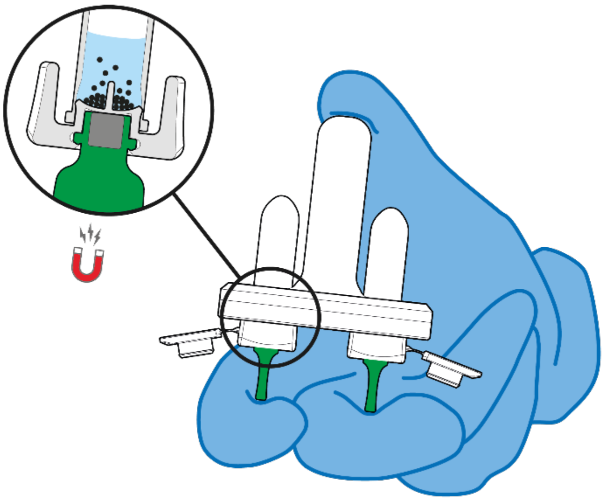

The Dual Tube Holder can also be used for Magnetic Bead collection. Immediately following each mixing step, insert the green magnets into both SmartLids and invert the entire Holder multiple times **until the liquid becomes clear**. As before, we recommend placing your fingers as shown to prevent the tubes and SmartLids from falling out.

DNA Extraction

Important: The following procedure will be illustrated for a **single sample.** For processing two samples simultaneously, all mixing and magnetic bead collection steps can be performed with the help of the included **Dual Tube Holder** as described in the previous section “**Processing Two Samples at a Time**” on **Page 8**.

Processing Two Samples Simultaneously is also demonstrated visually in the training video found at the following link (QR code to the right).

SAMPLE INPUT

1. Remove the sample tube lid and carefully set aside.

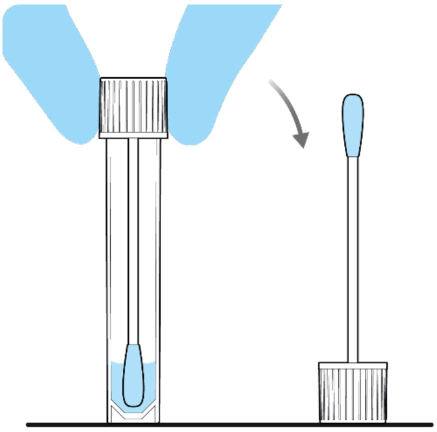

2. Open Tube A (Lysis) and set back into the tray.

   Use the included 400 µL exact volume pipette to collect sample and dispense into Tube A (Lysis).

   Discard pipette appropriately.

   Note: To operate the pipette, simply squeeze and release the upper bulb. Overflow is expected in the lower bulb as shown.

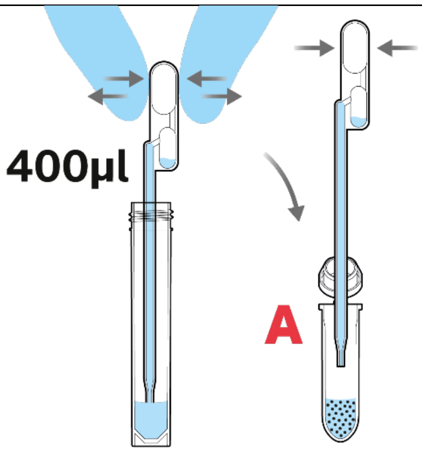

3. Insert SmartLid firmly into Tube A (Lysis).

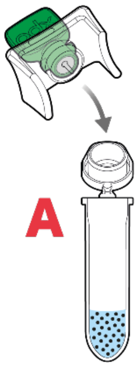

LYSIS

4. Remove the green magnet (twist counterclockwise to unlock).

Set the magnet into the preparation stand for later use.

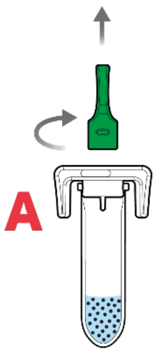

5. Use the vortex mixer to mix Tube A (Lysis) for 30 seconds.

Note: The companion app can assist with timing for each step of the Dragonfly process. (See Section “Companion Application” above.)

Access the app here on any device:

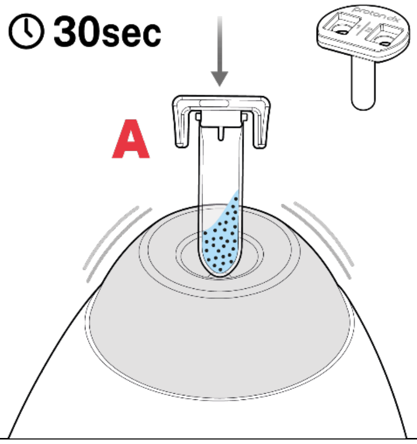

6. Insert the magnet back into the SmartLid and twist clockwise to lock.

Invert to collect the magnetic beads until the liquid is completely clear.

Pausing briefly between inversions can help speed up this process during the Lysis step.

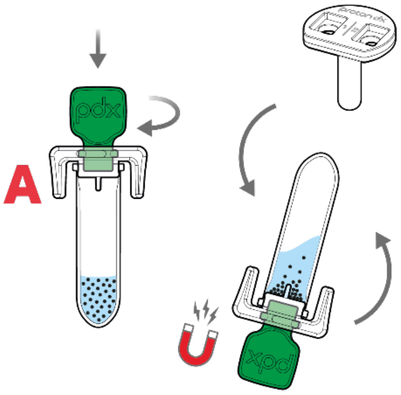

7. Once the liquid is clear and the magnetic beads are collected, remove the SmartLid from Tube A (Lysis) and insert into Tube B (Wash).

Note: The green magnet must remain inserted during this transfer process!

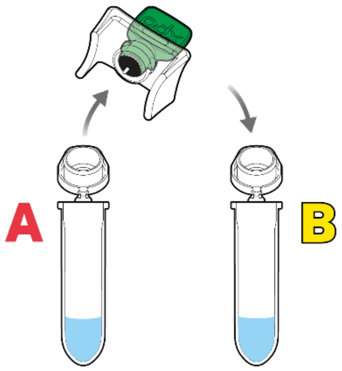

WASH

8. Remove the green magnet (twist to unlock).

Set the magnet into the preparation tray for later use.

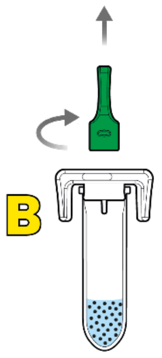

9. Use the vortex mixer to mix Tube B (Wash) for 30 seconds.

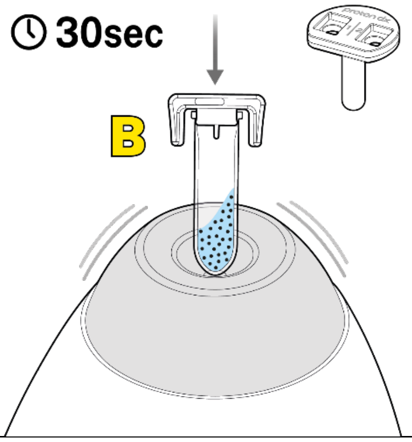

10. Insert the magnet back into the SmartLid and twist to lock.

Invert the tube to collect the magnetic beads until the liquid is completely clear.

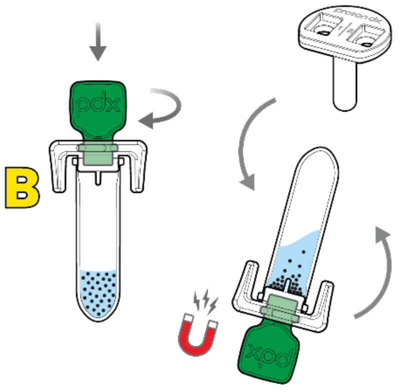

11. Remove SmartLid and place in the drying spot of the tray for 30 seconds as shown.

Note: The green magnet must remain inserted during the drying process!

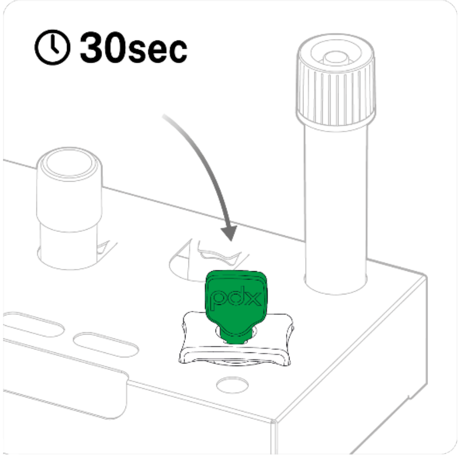

ELUTION

12. Insert SmartLid into Tube C (Elution) and remove the green magnet.

Set the green magnet into the preparation tray for later use.

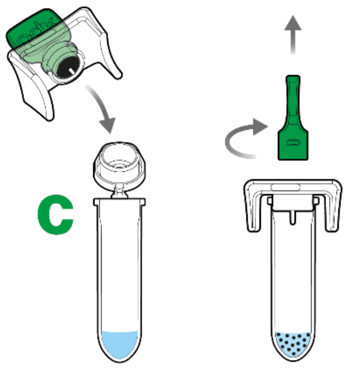

13. Use the vortex mixer to mix Tube C (Elution) for 30 seconds.

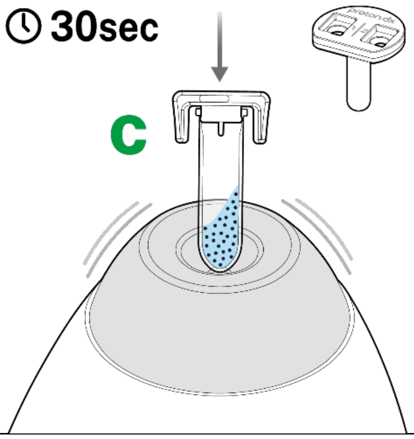

14. Insert the magnet back into the SmartLid and twist to lock.

Invert to collect the magnetic beads until the liquid is completely clear.

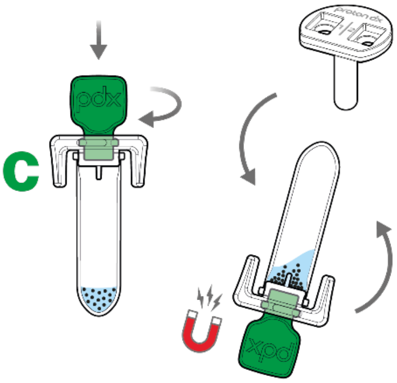

15. Flick the tube down to collect as much elution volume in the bottom as possible.

Remove the SmartLid and dispose of appropriately.

The sample is now purified and ready for loading into the Dragonfly Test Panel.

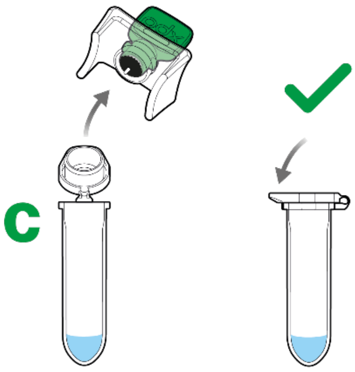

Detection: Test Panel Loading

Once the extractions are complete, testing should be performed using the Dragonfly OPXV / MPXV Test Panel and the dedicated Heat Block. Ensure the Heat Block is pre-heated to 63.5°C before incubation.

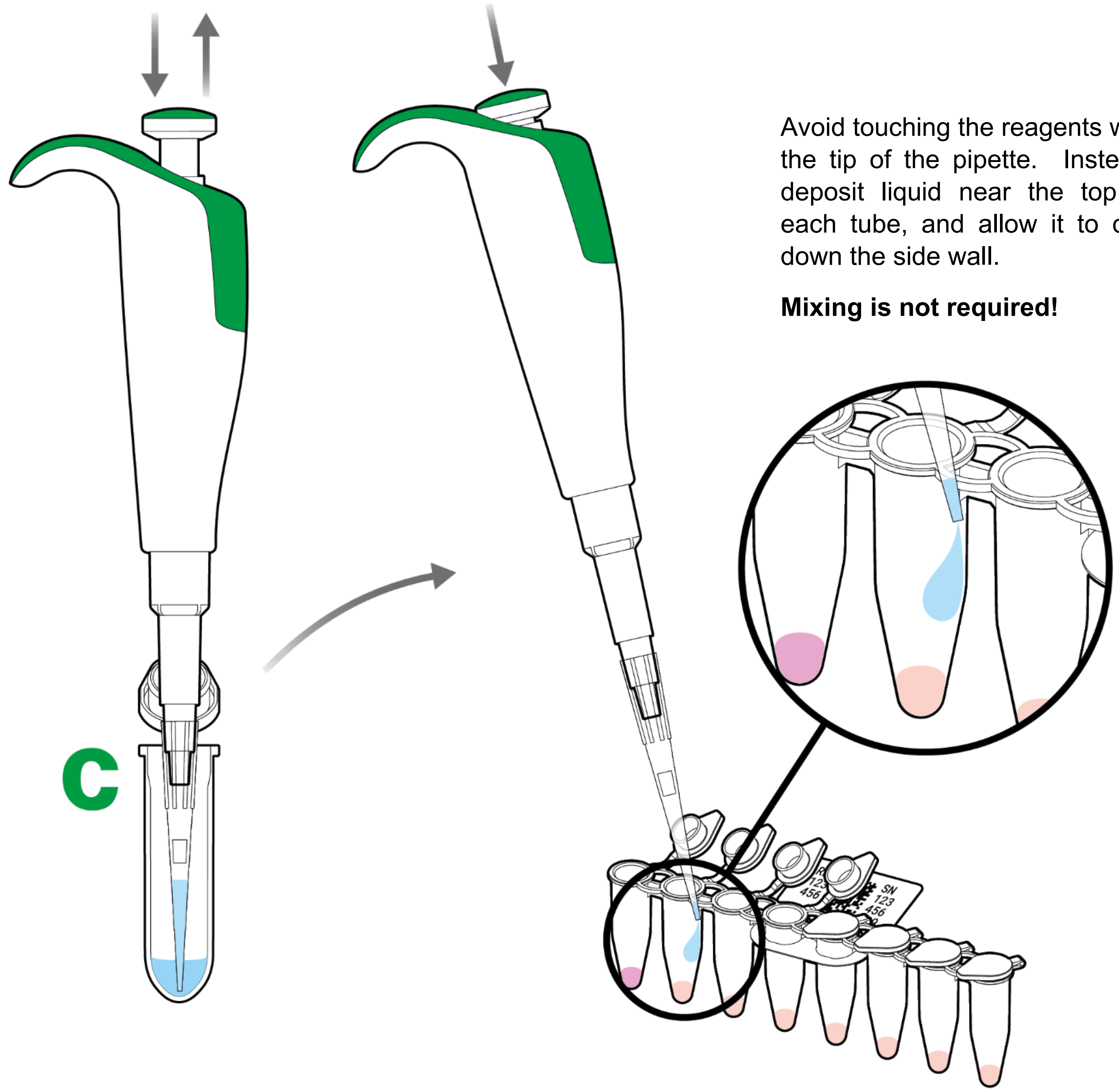

Avoid touching the reagents with the tip of the pipette. Instead, deposit liquid near the top of each tube, and allow it to drip down the side wall.

Mixing is not required!

1. Open the lids of the first 4 reaction tubes (from the left of the strip with the QR code facing you) of the OPXV / MPXV Test Panel and place the entire panel in the preparation stand for Sample 1.

2. Load a new disposable tip onto the reusable 20 µL pipette by pressing the pipette firmly into the yellow tip box.

3. Rehydrate reaction **tubes 1-4** with 20 µL from Tube C of **Sample 1's extraction**. Discard tip appropriately.

4. Firmly close the lids of the first four tubes.

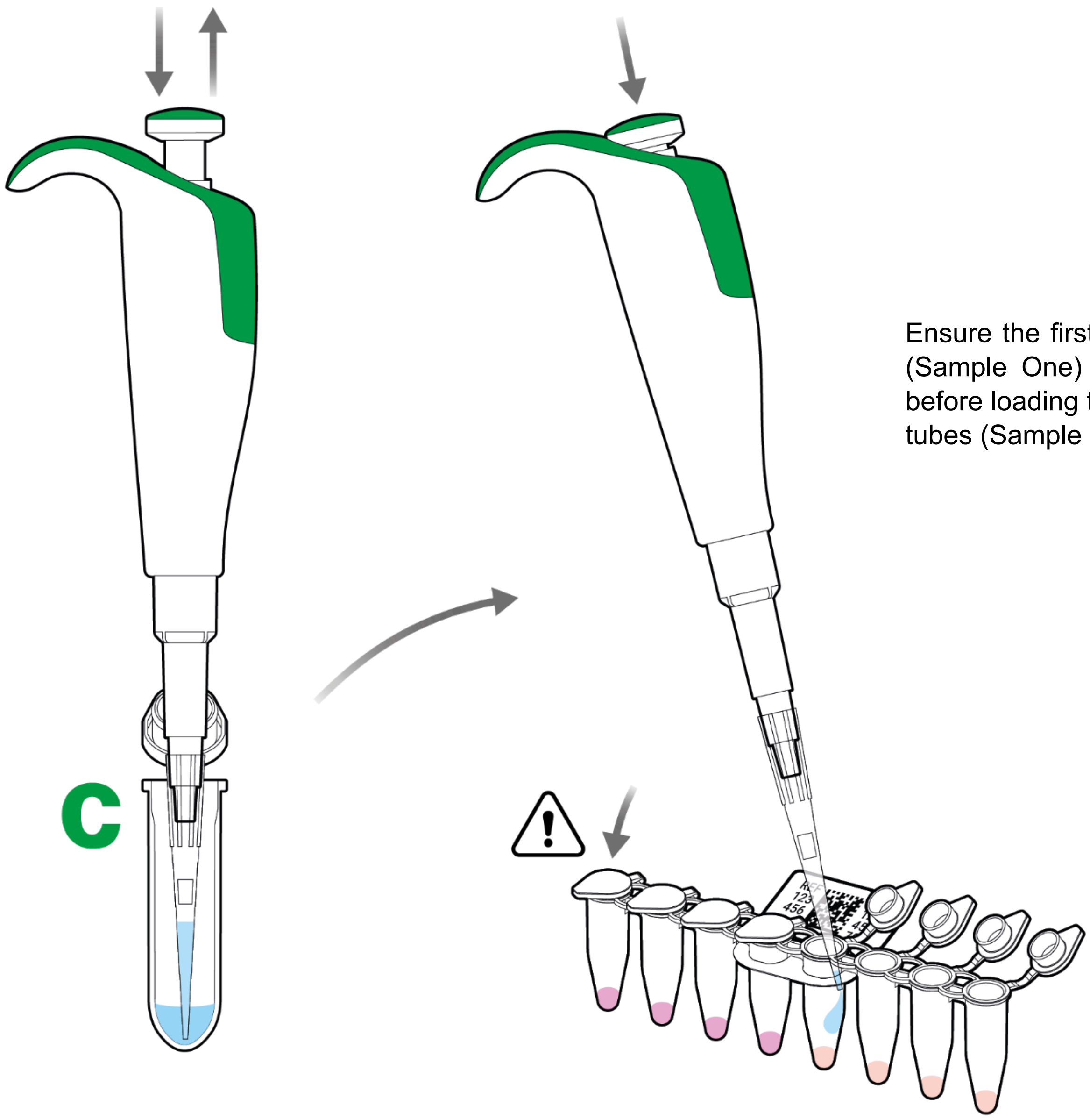

Ensure the first four tubes (Sample One) are closed before loading the last four tubes (Sample Two).

5. Open the lids of the last 4 reaction tubes (from the left of the strip with the QR code facing you) of the OPXV / MPXV Test Panel and place the entire panel in the preparation stand for Sample 2.

6. Load a new disposable tip onto the reusable 20 µL pipette by pressing firmly into the yellow tip box.

7. Rehydrate reaction **tubes 4-8** of the OPXV / MPXV Panel with 20 µL from Tube C of **Sample 2's extraction**. Discard tip appropriately.

8. Firmly close the last four tube lids and flick down the Dragonfly reaction tubes to make sure all the liquid sits at the bottom.

Flick the entire Test Panel downward to seat liquid in bases of each tube.

Important: Ensure all lids are closed securely!

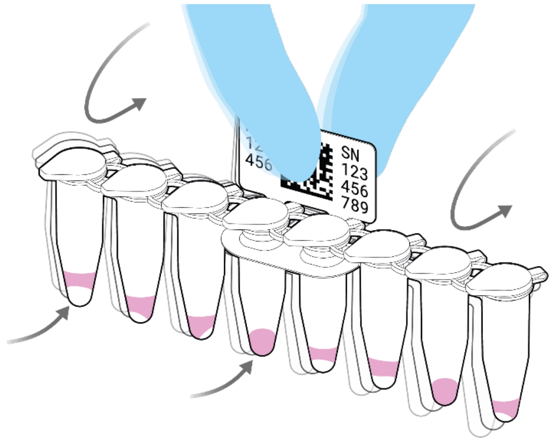

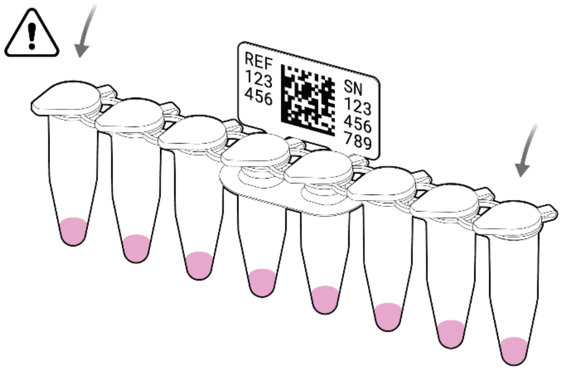

9. Transfer the Dragonfly Test Panel into the pre-heated ProtonDx Heat Block. Ensure that the heater has reached 63.5°C prior to this step, and check that all the lids are securely closed.

10. Incubate the test panel for 45 minutes, making sure to select the same row in both the Heat Block Companion App as shown below:

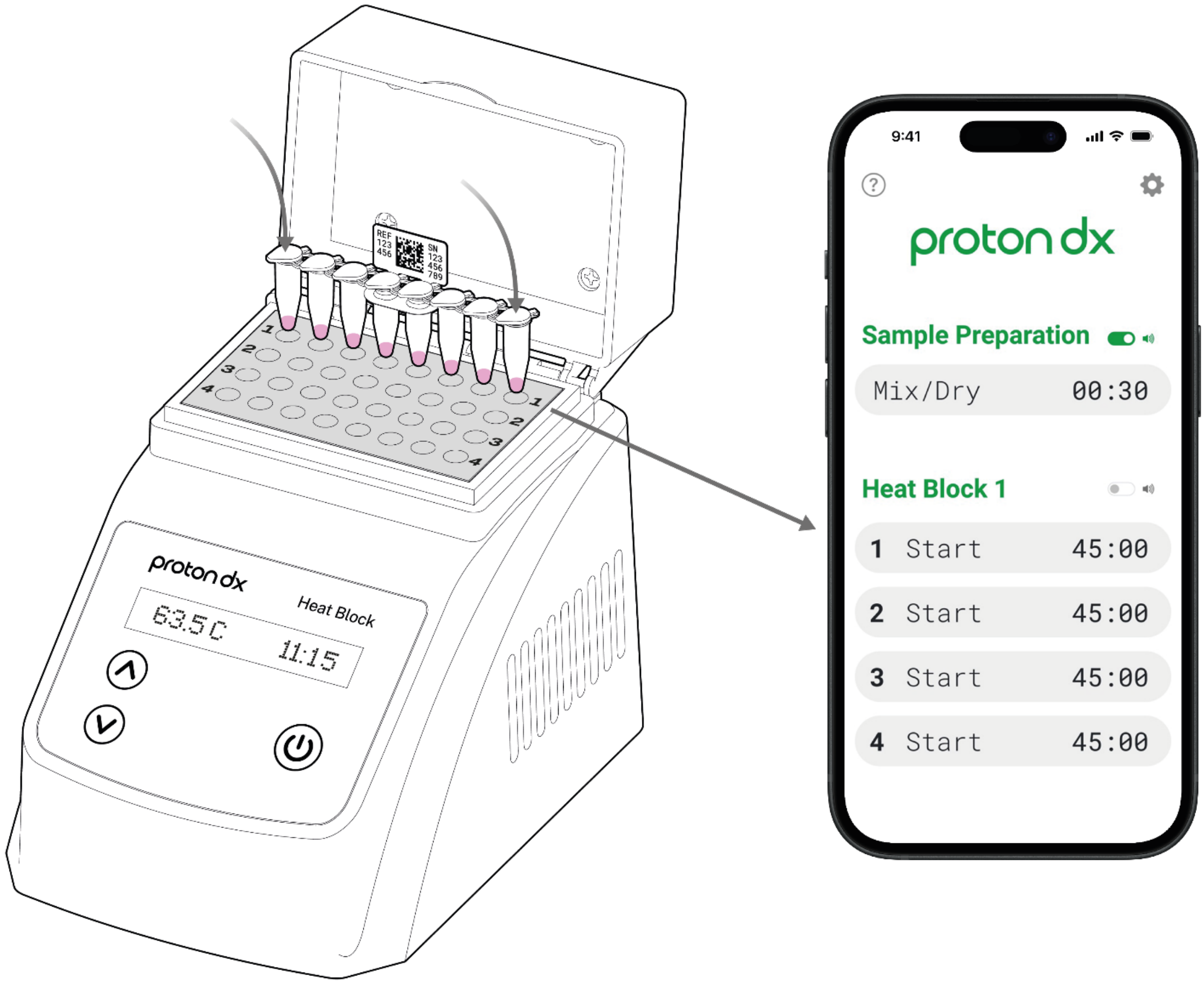

11. After the time has elapsed, remove the Dragonfly test panel from the heater (pulling upward from the attached tag) and place it in the results card with the QR code facing toward you as shown below:

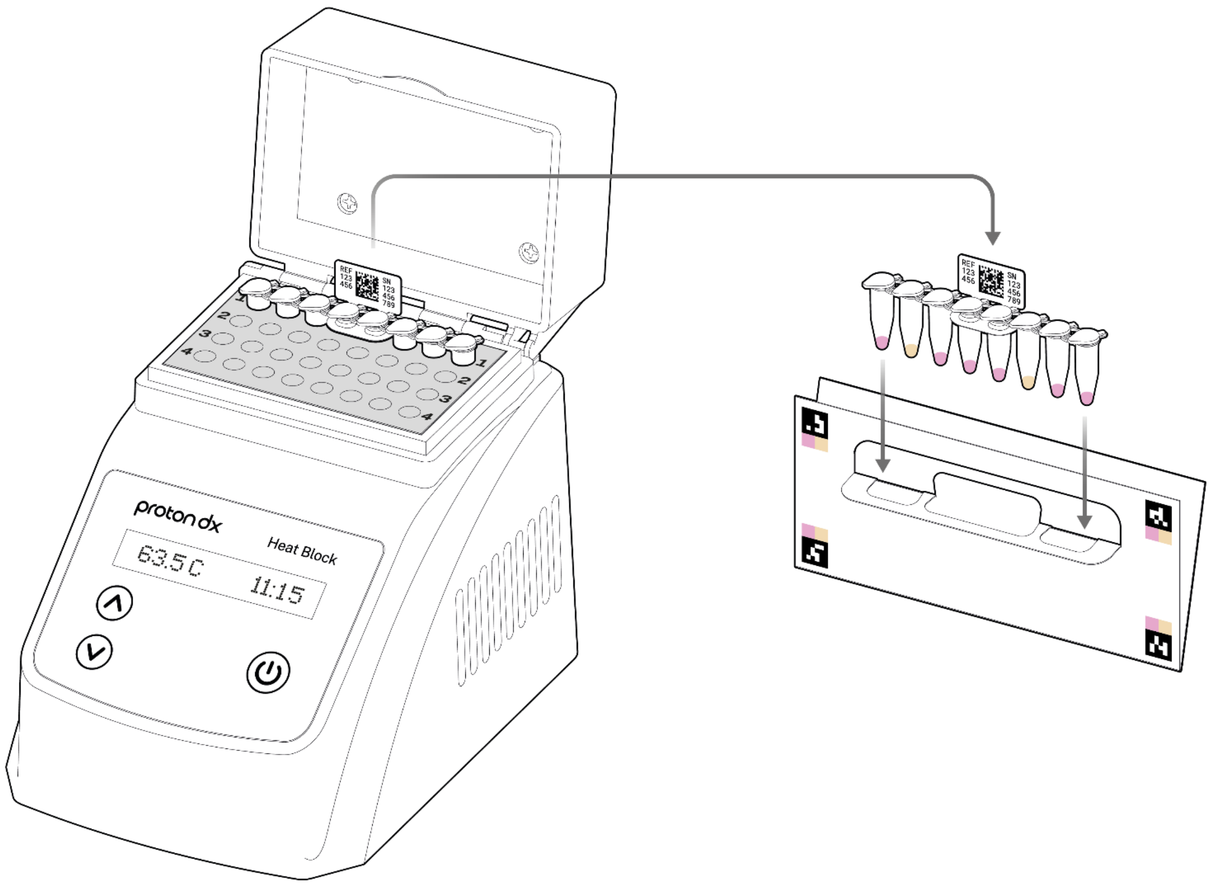

Result Interpretation

Important: Always ensure the tube lids remain closed.

A pen may be used to mark your selections and write additional details onto the card. Write the Sample ID, Date, and operator's name on the card, along with the results of the test.

The Dragonfly OPXV / MPXV Test Panel can provide results for up to two patient samples simultaneously. For each result, there are two control reactions and two target reactions.

Outcomes will be either Pink or Yellow.

- **Tube 1** is a colour reference and **must stay pink**. If it turns yellow, the test for that sample is invalid.
- **Tube 2** is an internal control and **must turn yellow**. If it remains pink, the test for that sample is invalid.

If the controls all match the expected colours, the test is valid. ✓

- **Tube 3** is the OPXV reaction for Sample 1. If it is yellow, then the result is positive. If it remains pink, then the result is negative.
- **Tube 4** is the MPXV reaction for Sample 1. If it is yellow, then the result is positive. If it remains pink, then the result is negative.

This order is repeated for the second half of the test panel (tubes 4-8), but for Sample 2.

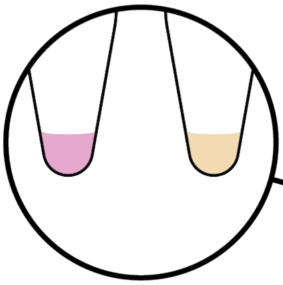

Results will be either pink (negative) or yellow (positive).

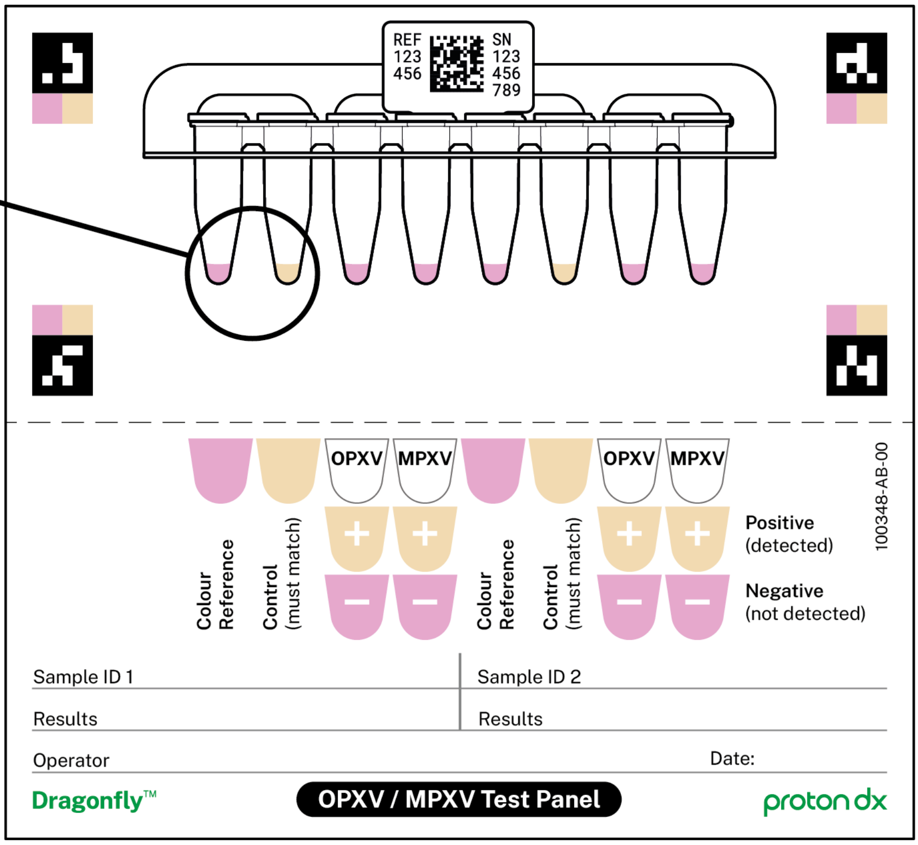

Use the bottom half of the Result Card to record outcomes and track operators.

6. Troubleshooting

Problem	Possible Cause	Suggested Solution
Heat Block is not turning on or warming up	Incomplete set up of Heat Block	The ProtonDx Heat Block requires two steps to turn on. First, the switch on the back of the device must be flipped to the "on" position. Second, the power button on the front of the device must be pressed once. An audible beep will indicate that both steps have been completed, and the displayed temperature should start flashing in increasing.
	Loose power connector	Ensure connections between the outlet cord and power brick, as well as the DC connector and the Heat Block, are both securely and fully inserted.
Disposable Exact Volume Pipette is not functioning	Puncture in plastic wall of Exact Volume Pipette	Use the secondary Exact Volume Pipette as provided in every Sample Preparation Kit.

Dried reagents are stuck near the caps of the test panel tubes	Movement during transport	Gently tap the bases of the tubes against a hard surface. Note, it is not required to fully seat the reagents in the base of each tube. We recommend simply ensuring they are in the bottom half of each tube, to prevent accidental contact with the pipette tip during loading.
Test Panel reagents resuspend already yellow	Expired or damaged test panel	Please do not use the Test Panel. If more than one Test Panel has this problem, and the batch is not expired, please contact technical support.
Companion App alarms do not make noise	Device volume off/low, device locked/asleep, application not running in foreground	The Dragonfly Companion App runs in a web browser, and thus must always be actively running in the foreground for the alarms to sound. Note, however, that the timers will still keep an accurate track of time regardless, even if the device falls asleep.
Invalid Test Panel Controls	Incorrect incubation temperature	Check to ensure that the Heat Block display reads 63.5 C. If not, ensure the Heat Block is on (see beginning of troubleshooting section).
	Incomplete incubation	Make sure the correct Test Panel was removed from the Heat Block, and if so, that the Test Panel was not removed from the Heat Block before its timer expired.
	Expired or damaged reagents	Check Test Panel expiration date. If expired, discard batch. If still in-date, select a second Test Panel and repeat the extraction and detection process. Note, each Sample Tube contains enough sample to complete two full extractions.

7. Technical Support

Answers to workflow questions may be found by watching the videos and reviewing the video linked to the QR Code below:
