# Supplementary material for: Portable molecular diagnostic platform for rapid point-of-care detection of mpox and other diseases
Source: Nat Commun. 2025 Mar 24;16:2875. doi: 10.1038/s41467-025-57647-3 (PMC11933461; doi:10.1038/s41467-025-57647-3)
Supplement: Supplementary file 2 — Description of Additional Supplementary Files [file 41467_2025_57647_MOESM2_ESM.pdf]

Description of Additional Supplementary Files

Supplementary Data 1 -

Raw data Dragonfly and PCR (Tab 1): Raw data of the samples tested as part of the clinical validation of Dragonfly Skin Infection Viral Test Panel. All the anonymised samples are included in rows, with the corresponding data obtained with Dragonfly (detected or not detected) and PCR (C_t values).

OPXV_optimisation (Tab 2): Raw data of the evaluation of assay candidates to detect OPXV. Each table includes the name of the assay, target, the C_t values (minutes), concentration of the target gBlock in copies/reaction, mean C_t (minutes) and standard deviation (minutes). The sequences of each of the primers is specified in the corresponding tables. In the case where the name of the assay includes 2uM, 2.5uM etc., this is the final concentration of FIP and BIP used per reaction.

MPXV_optimisation (Tab 3): Raw data of the evaluation of assay candidates to detect MPXV. Each table includes the name of the assay, target, the C_t values (minutes), concentration of the target gBlock in copies/reaction, mean C_t (minutes) and standard deviation (minutes). The sequences of each of the primers is specified in the corresponding tables. In the case where the name of the assay includes 2uM, 2.5uM etc., this is the final concentration of FIP and BIP used per reaction.

VZV_optimisation (Tab 4): Raw data of the evaluation of assay candidates to detect VZV. Each table includes the name of the assay, target, the C_t values (minutes), concentration of the target gBlock in copies/reaction, mean C_t (minutes) and standard deviation (minutes). The sequences of each of the primers is specified in the corresponding tables. In the case where the name of the assay includes 2uM, 2.5uM etc., this is the final concentration of FIP and BIP used per reaction.

HSV_optimisation (Tab 5): Raw data of the evaluation of assay candidates to detect HSV1 and HSV2. Each table includes the name of the assay, target, the C_t values (minutes), concentration of the target gBlock in copies/reaction, mean C_t (minutes) and standard deviation (minutes). The sequences of each of the primers is specified in the corresponding tables. In the case where the name of the assay includes 2uM, 2.5uM etc., this is the final concentration of FIP and BIP used per reaction.

Standard curves (Tab 6): Data used to plot the standard curves in Figure S1. Each table includes the name of the assay, target concentration of the gBlock in copies/reaction, mean C_t (minutes) and standard deviation (minutes).

Methods (Tab 7): Catalogue number and company of the reagents used for the LAMP master mix in this study.
